# Integrated microbial and transcriptomic profiling reveals distinct correlations in atrophic gastritis associated with liver cirrhosis

**DOI:** 10.3389/fcimb.2026.1893991

**Published:** 2026-08-21

**Authors:** Yiqing Zhu, Guoming Zhang, Ruiguang Ma, Qian Li, Huayu Dai, Zhouyue Li, Lixiang Li, Zhen Li

**Affiliations:** 1 Department of Gastroenterology, Qilu Hospital of Shandong University, Jinan, Shandong, China; 2 Shandong Provincial Clinical Research Center for Digestive Disease, Jinan, Shandong, China; 3 Laboratory of Translational Gastroenterology, Qilu Hospital of Shandong University, Jinan, Shandong, China; 4 Robot Engineering Laboratory for Precise Diagnosis and Therapy of GI Tumor, Qilu Hospital of Shandong University, Jinan, Shandong, China; 5 Department of Gastroenterology, The Affiliated Hospital of Qingdao University, Qingdao, Shandong, China; 6 Department of Gastroenterology, Binzhou Medical University Hospital, Binzhou, Shandong, China

**Keywords:** chronic atrophic gastritis, liver cirrhosis, machine learning, microbiota, transcriptome

## Abstract

**Objective:**

To explore the alterations in the microbiota and transcriptome of patients with chronic atrophic gastritis (CAG) and liver cirrhosis (LC) and to develop robust diagnostic models.

**Methods:**

16S ribosomal RNA (rRNA) amplicon sequencing was performed on saliva and gastric mucosal samples, and transcriptome sequencing was performed on gastric mucosal samples from the study participants.

**Results:**

*Veillonella, Ruminococcus_gnavus_group*, *Olsenella*, and other genera were enriched in the saliva of LC patients with CAG (Cirrhosis A) compared with both LC patients without CAG (Cirrhosis N) and controls without LC (Control), while *Lautropia* was reduced (LDA > 2.0, adjusted *P*-value < 0.05). Moreover, in the independent test set, the combination of *Cardiobacterium* and *Olsenella* classified Cirrhosis A subjects from Cirrhosis N subjects with an area under the curve (AUC) of 0.89, and classified Cirrhosis A subjects from Control subjects with an AUC of 0.84. An eight-genera panel achieved an AUC of 0.92 for classifying Cirrhosis A from Control. In the gastric mucosa, *ABCG5*, *ITGA2*, *MYB*, and other genes were upregulated, whereas EPOR, *CHAD, IGFALS*, and other genes were downregulated in Cirrhosis A compared with both Cirrhosis N and CAG patients without LC (Control A) (adjusted *P*-value < 0.05). The overlapping differentially expressed genes were enriched, including in the PI3K-Akt signaling pathway, bile secretion, cholesterol metabolism, PPAR signaling, and steroid biosynthesis pathways. In Cirrhosis A, correlation analysis revealed that increased *Aggregatibacter* was positively correlated with *TTC22* and *PRSS3*, whereas increased *Allobaculum* was positively correlated with *TFAP2B*.

**Conclusion:**

LC is associated with distinct microbial and transcriptomic reprogramming in CAG, with enrichment of the PI3K-Akt signaling and bile secretion pathways, suggesting their possible involvement in cirrhosis-associated gastric mucosal changes and warranting further investigation.

## Introduction

Chronic atrophic gastritis (CAG) and liver cirrhosis (LC) represent significant chronic disorders within the digestive system, each associated with substantial morbidity and mortality worldwide (Tan and Yeoh, 2015; Ginès et al., 2021). CAG is typified by mucosal atrophy, the prominence of submucosal vessels, and the presence of mucosal nodules (Tan and Yeoh, 2015). This condition marks the initial stage of a multistep precancerous process, progressing to advanced stages such as gastric intestinal metaplasia (GIM), dysplasia, and culminating in gastric adenocarcinoma (Correa et al., 1975). In contrast, LC typically arises as a consequence of chronic inflammation, with the progressive replacement of normal liver parenchyma by fibrotic tissue and regenerative nodules, a pathological change that culminates in portal hypertension (Ginès et al., 2021). Collectively, these conditions contribute significantly to the global disease burden (Huang et al., 2023; Liu et al., 2024).

Numerous studies have documented a variety of gastric mucosal abnormalities in patients with LC and portal hypertension, including inflammatory-like changes (such as edema, erythema, granularity, and friability), erosive gastritis, gastric ulcers, and GIM (Ibrişim et al., 2008; Norman and Pirlich, 2008; Mohamed et al., 2018). In patients with LC and portal hypertension, the most frequent endoscopic findings include snake skin (71%), scarlatina rash (70%), and petechia (60%) (Vigneri et al., 1991). Furthermore, the enrichment of specific pathogenic bacteria in the stomach may exacerbate gastric inflammation or contribute to the development of gastric cancer (GC) (Sung et al., 2020). However, uncertainties persist regarding the characterization of gastric microbiota in atrophic gastritis with LC and their relationship with gastric mucosal abnormalities.

Patients with advanced stages of CAG require systematic follow-up using high-quality endoscopy (Pimentel-Nunes et al., 2019). In contemporary clinical practice, gastroscopy coupled with tissue biopsy remains the cornerstone for diagnosing CAG (Schaub et al., 2004; Yu et al., 2011; Rodriguez-Castro et al., 2018; Hao et al., 2020). Nevertheless, the invasive nature of endoscopy has several limitations. This is particularly problematic for patients with LC, who are predisposed to complications such as bleeding, nausea, and vomiting (Abd-Elsalam et al., 2022; Gai et al., 2022). Given these constraints, developing a non-invasive diagnostic model for CAG in LC patients is imperative for improving both diagnostic accuracy and patient comfort.

In this study, we sought to further characterize the spectrum of atrophic gastritis in the setting of LC. Specifically, we investigated alterations in microbiota and transcriptional activity in atrophic gastritis among LC patients, elucidating the transition mechanism from typical to atrophic gastritis in patients with LC. Ultimately, we aimed to develop a non-invasive model to distinguish atrophic gastritis and its conventional counterpart, particularly among patients with LC.

## Methods

### Subject recruitment

We conducted an observational study involving 25 patients with LC and CAG (Cirrhosis A), 48 patients with LC without CAG (Cirrhosis N), and 51 controls without LC (Control) from August 2021 to July 2022 at the Qilu Hospital of Shandong University, Jinan, Shandong Province. LC diagnosis adhered to international guidelines, encompassing clinical symptoms, physical signs, radiological examinations, laboratory tests, medical history, and cirrhosis-associated complications of chronic liver disease (Rodriguez-Castro et al., 2018). CAG diagnosis was confirmed by pathological examination. Participants older than 18 years who provided signed informed consent were included in the study. Exclusion criteria comprised: (I) cardiovascular disease, diabetes, inflammatory bowel disease (IBD), or mental disorders; and (II) recent use of proton pump inhibitors (PPI), antibiotics, hormones, immunosuppressants, or chemotherapy drugs within one month before enrollment. The study was conducted in accordance with the Declaration of Helsinki and was approved by the Ethics Committee of Qilu Hospital of Shandong University (KYLL-202107-152). All procedures was performed in accordance with relevant guidelines. Written informed consent was obtained from each patient before enrollment.

### Sample collection and processing

All subjects were asked to rinse their mouths with normal saline according to published protocols (Ghannoum et al., 2010). The rinse was discarded, and 3–5 mL of saliva was collected in cryopreservation tubes and promptly stored at –80 °C until 16S ribosomal RNA (rRNA) amplicon sequencing. Two experienced endoscopists with at least five years of experience performed high-definition gastroscopy (Pentax EG29-i10, Pentax, Tokyo, Japan) on all participants. Gastroscopic examination involved scrutinizing each mucosal segment from the gastric antrum to the body of the stomach, followed by a rapid urease test (RUT). *H. pylori* status was further confirmed by modified Giemsa staining on gastric mucosal sections, and subjects were classified as positive if either the RUT result or modified Giemsa staining result was positive. Two gastric tissue fragments were obtained using 2.2-mm sterile biopsy forceps and promptly stored at –80 °C. One fragment was designated for 16S rRNA sequencing, while the other was preserved in RNALater for subsequent transcriptomics analysis.

### DNA extraction, 16S rRNA amplicon sequencing, and data analysis

According to the manufacturer’s instructions, total microbial genomic DNA was extracted from saliva and gastric mucosal samples using the E.Z.N.A.^®^ soil DNA Kit (Omega Bio-tek, Norcross, GA, USA). The quality and concentration of DNA were determined by 1.0% agarose gel electrophoresis and using a NanoDrop^®^ ND-2000 spectrophotometer (Thermo Scientific Inc., USA). Subsequently, the hypervariable V3–V4 region of the bacterial 16S rRNA gene was amplified using primer pairs 338F (5’-ACTCCTACGGGAGGCAGCAG-3’) and 806R (5’-GGACTACHVGGGTWTCTAAT-3’), facilitated by an ABI GeneAmp^®^ 9700 polymerase chain reaction (PCR) thermocycler based in California, USA.

Paired-end reads were consolidated into sequence tags, adhering to their overlapping characteristics and subject to rigorous quality control measures using the UPARSE pipeline. This step excluded subpar reads and chimeric sequences, using vsearch software (with parameters set to maxee = 3 and minlength = 370) for effective filtration. Upon dereplication, the remaining clean reads were clustered into distinct operational taxonomic units (OTUs) using a 97% similarity threshold. Representative reads from 7,219 OTUs were aligned against the Ribosomal Database Project, version 18, to ascertain taxonomic identities, requiring an 80% similarity match to species entries within the reference database, thereby providing insights into the microbiome composition. Bioinformatic microbiome analysis was carried out using the Majorbio Cloud platform (https://cloud.majorbio.com). Detailed sequencing quality control metrics for each sample are provided in **Supplementary Table 10**.

To assess the potential confounding effects of clinical factors on microbial community variation, we performed PERMANOVA using the adonis2 function in the vegan package. Age, sex, body mass index (BMI), smoking history, alcohol consumption, and *H. pylori* status were included as covariates in all comparisons. For analyses restricted to the cirrhotic population, including Cirrhosis A and Cirrhosis N, Child–Pugh class, model for end-stage liver disease (MELD) score, cirrhosis etiology, esophageal varices grade, and portal vein diameter (PVD) were additionally included. To build the diagnostic model, all samples were randomly divided into a 70% training set and a 30% independent test set. In the training set, differential genera were identified using LEfSe and Wilcoxon rank-sum tests, followed by random forest (RF) classification with 5-fold cross-validation for hyperparameter optimization (mtry = √p, ntree = 100). The final model was validated on the independent test set, and the area under the curve (AUC) was calculated using receiver operating characteristic (ROC) curves.

### RNA extraction, transcriptome sequencing, and data analysis

Total RNA of gastric tissue was extracted using TRIzol^®^ Reagent according to the manufacturer’s instructions. RNA quality was assessed with a 5300 Bioanalyser and quantified using a NanoDrop ND-2000. High-quality RNA (OD260/280 = 1.8–2.2, RIN ≥ 6.5) was used for library construction. The RNA-seq library was prepared using Illumina^®^ Stranded mRNA Prep. Briefly, messenger RNA was isolated using the polyA selection method with oligo(dT) beads and was then first fragmented using fragmentation buffer. Next, double-stranded cDNA was synthesized using a SuperScript double-stranded cDNA synthesis kit (Invitrogen, CA, USA) with random hexamer primers (Illumina). Then, the synthesized cDNA was subjected to end-repair, phosphorylation, and ‘A’ base addition according to Illumina’s library construction protocol. Libraries were size-selected for 300-bp fragments and PCR-amplified. After quantification using Qubit 4.0, the paired-end RNA-seq sequencing library was sequenced with the NovaSeq Xplus sequencer (Illumina, San Diego, CA, USA).

The raw paired-end reads were trimmed and quality controlled by fastp (Chen et al., 2018) with default parameters. The mapped reads from each sample were assembled using StringTie (Pertea et al., 2015) with a reference-based approach. Differential expression was analyzed using DESeq2 (Love et al., 2014) or DEGseq (Wang et al., 2010). Genes with |log2FC| ≥ 1 and a false discovery rate (FDR) ≤ 0.05 were considered significant. Gene Ontology (GO) and Kyoto Encyclopedia of Genes and Genomes (KEGG) enrichment analyses were performed using Goatools and KOBAS (Xie et al., 2011). RSEM software was used to quantify gene and transcript expression levels. Bioinformatic transcriptome analysis was carried out using the Majorbio Cloud platform (https://cloud.majorbio.com).

## Results

### Alterations of salivary microbiota in Cirrhosis A compared with cirrhosis N and Control

**Figure 1:**
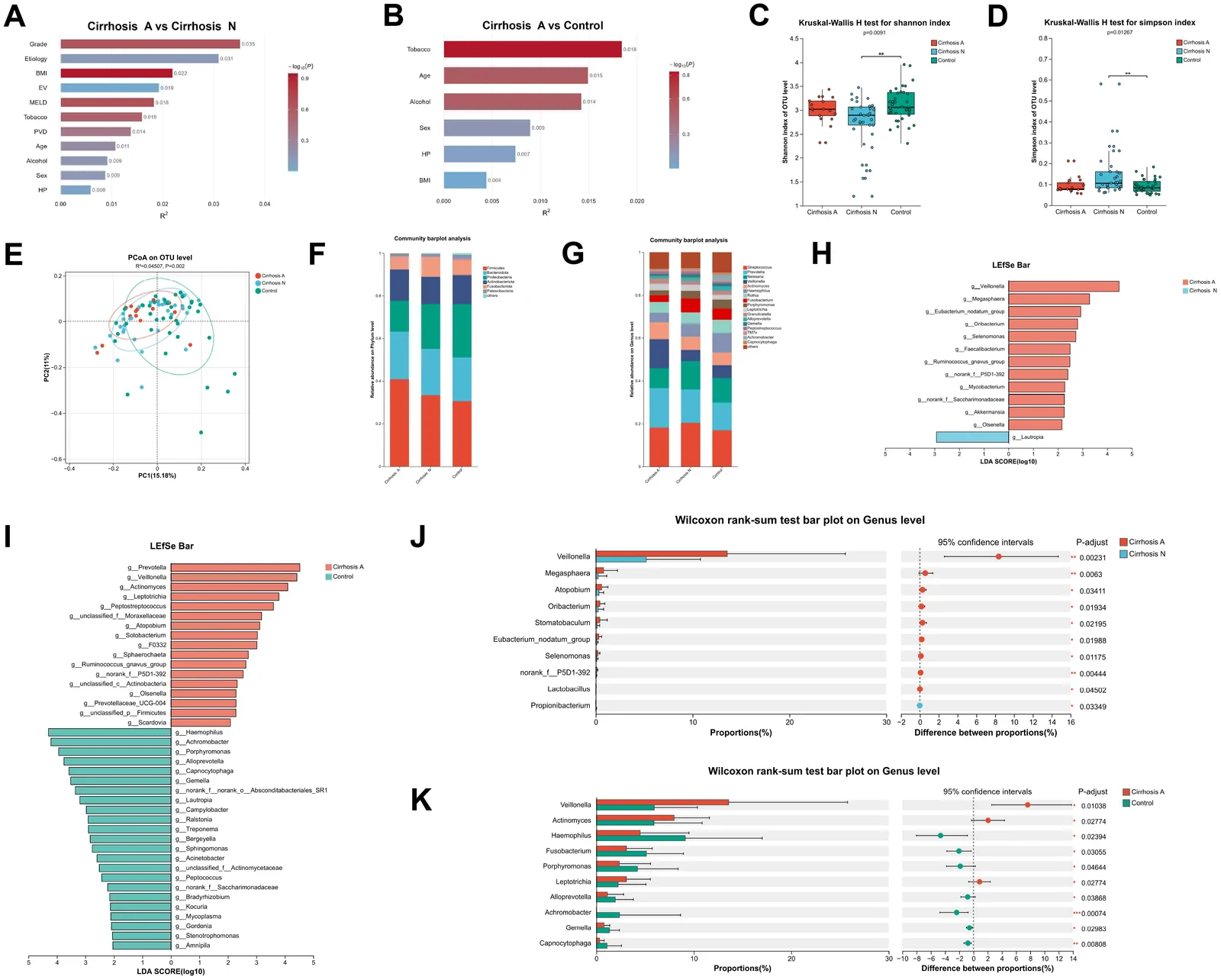
Alterations in salivary microbiota in cirrhosis A compared with cirrhosis N and control. **(A)** PERMANOVA analysis of salivary microbiota between Cirrhosis A and Cirrhosis N, with liver-related (Child–Pugh, MELD, etiology, varices, PVD) and non-liver-related covariates (age, sex, BMI, smoking, alcohol, and *H. pylori*) included. **(B)** PERMANOVA analysis of salivary microbiota between Cirrhosis A and Control, with non-liver-related covariates (age, sex, BMI, smoking, alcohol, and *H. pylori*) included. All covariates had P > 0.05. **(C, D)** The Shannon and Simpson indices were used to evaluate the salivary microbial diversity of Cirrhosis A, Cirrhosis N, and Control. **(E)** PCoA of weighted UniFrac distance demonstrated that the three groups showed three distinct clusters. **(F, G)** Relative microbial abundance in the saliva of the three groups at the phylum and genus levels. **(H)** Differential genera between Cirrhosis A and Cirrhosis N identified by LEfSe analysis (LDA > 2.0, Q < 0.05). **(I)** Differential genera between Cirrhosis A and Control identified by LEfSe analysis (LDA > 2.0, Q < 0.05). **(J)** Differential genera between Cirrhosis A and Cirrhosis N identified by the Wilcoxon rank-sum test (adjusted *P*-value < 0.05). **(K)** Differential genera between Cirrhosis A and Control identified by the Wilcoxon rank-sum test (adjusted *P*-value < 0.05). *, q < 0.05, and **, q < 0.01.

As shown in **Supplementary Table 1**, no significant differences were observed across any clinical parameters between the compared groups (all *P* > 0.05). For the salivary microbiota, PERMANOVA analysis revealed that all included clinical covariates showed no significant effects in either the comparison between Cirrhosis A and Cirrhosis N (**Figure 1A**) or the comparison between Cirrhosis A and Control (**Figure 1B**, all *P* > 0.05), with consistently low *R^2^* values ranging from 0.004 to 0.035, indicating that these factors explained only a minimal fraction of the microbial variation. We first measured the microbial alpha-diversity of the saliva using different methodologies. The Shannon and Simpson indices revealed that the microbial community richness and diversity in the saliva of Cirrhosis N were significantly lower than those of Control (**Figures 1C, D**). Analysis of beta-diversity using principal coordinates analysis (PCoA) revealed differences in salivary bacterial communities among Cirrhosis A, Cirrhosis N, and Control (*P*-value = 0.002, **Figure 1E**). At the phylum level, the top five pancreatic bacteria identified in the saliva of Cirrhosis A, Cirrhosis N, and Control were *Firmicutes*, *Bacteroidota*, *Proteobacteria*, *Actinobacteriota*, and *Fusobacteriota*, indicating a broad phyla-level similarity (**Figure 1F**). However, at the genus level, the top five genera in the saliva of Cirrhosis A were *Prevotella*, *Streptococcus*, *Veillonella*, *Neisseria*, and *Actinomyces*. *Streptococcus*, *Prevotella*, *Neisseria*, *Fusobacterium*, and *Actinomyces* were the top five genera detected in the saliva of Cirrhosis N. The top five genera in the saliva of Control were *Streptococcus*, *Prevotella*, *Neisseria*, *Haemophilus*, and *Rothia* (**Figure 1G**). Linear discriminant analysis (LDA) effect size (LEfSe) (LDA > 2.0, adjusted *P*-value < 0.05) found that *Veillonella*, *Ruminococcus_gnavus_group*, *g:norank_f:P5D1-392*, and *Olsenella* were enriched in the saliva of Cirrhosis A compared with both Cirrhosis N (**Figure 1H**) and Control (**Figure 1I**), while *Lautropia* was reduced. After *P*-value correction by the Benjamini–Hochberg (BH) method, the Wilcoxon rank-sum test showed that *Veillonella*, *Atopobium*, *g:norank_f:P5D1-392*, *Lactobacillus*, and *g:unclassified_p:Firmicutes* were increased in the saliva of Cirrhosis A compared with both Cirrhosis N (adjusted *P*-value < 0.05, **Figure 1J**, **Supplementary Table 2**) and Control (**Figure 1K**, **Supplementary Table 2**), while *Simonsiella* was decreased.

### Salivary microbial genera as cirrhosis A diagnostic markers

**Figure 2:**
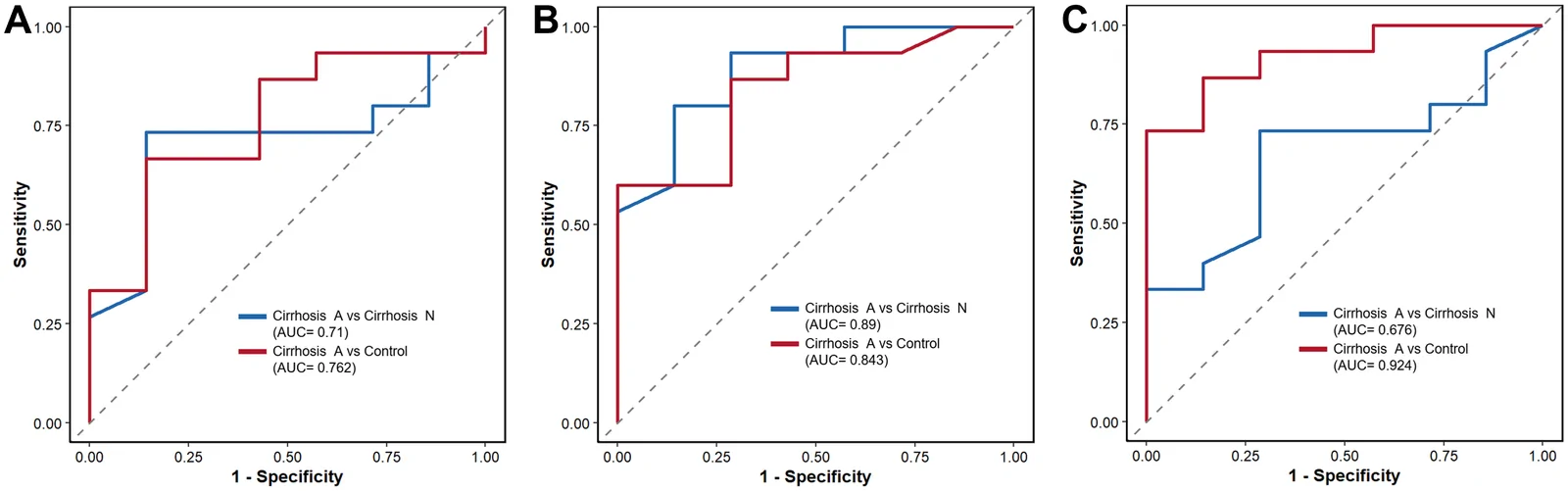
Microbial markers for discrimination of cirrhosis A from cirrhosis N and control. **(A)** Receiver operating characteristic curve (ROC) for the six differential genera markers discriminating Cirrhosis A from Cirrhosis N and Control. **(B)** ROC for two genera markers discriminating Cirrhosis A from Cirrhosis N and Control. **(C)** ROC for eight genera markers discriminating Cirrhosis A from Control and Control.

To validate these differential microbial candidates in the saliva selected from the above methods, including *Veillonella*, *Ruminococcus_gnavus_group*, *g:norank_f:P5D1-392*, *Olsenella*, *Lautropia*, and *g:unclassified_p:Firmicutes*, we next built multispecies classifiers in the training set based on these genera using RF and five-fold cross-validation. In the independent test set, this combination distinguished Cirrhosis A from Cirrhosis N with an AUC of 0.71 and Cirrhosis A from Control with an AUC of 0.76 (**Figure 2A**). Furthermore, the combination of *Cardiobacterium* and *Olsenella* increased the AUC to 0.89 for discriminating Cirrhosis A from Cirrhosis N and achieved an AUC of 0.84 for distinguishing Cirrhosis A from Control (**Figure 2B**). For classifying Cirrhosis A from Control, a panel of eight bacterial genera (*Achromobacter*, *Actinomyces*, *Atopobium*, *Neisseria*, *Olsenella*, *Sphingomonas*, *Veillonella*, *and g:norank_f:Saccharimonadaceae*) achieved a higher AUC of 0.92 (**Figure 2C**).

### Alterations of gastric microbiota in cirrhosis A compared with cirrhosis N and control

**Figure 3:**
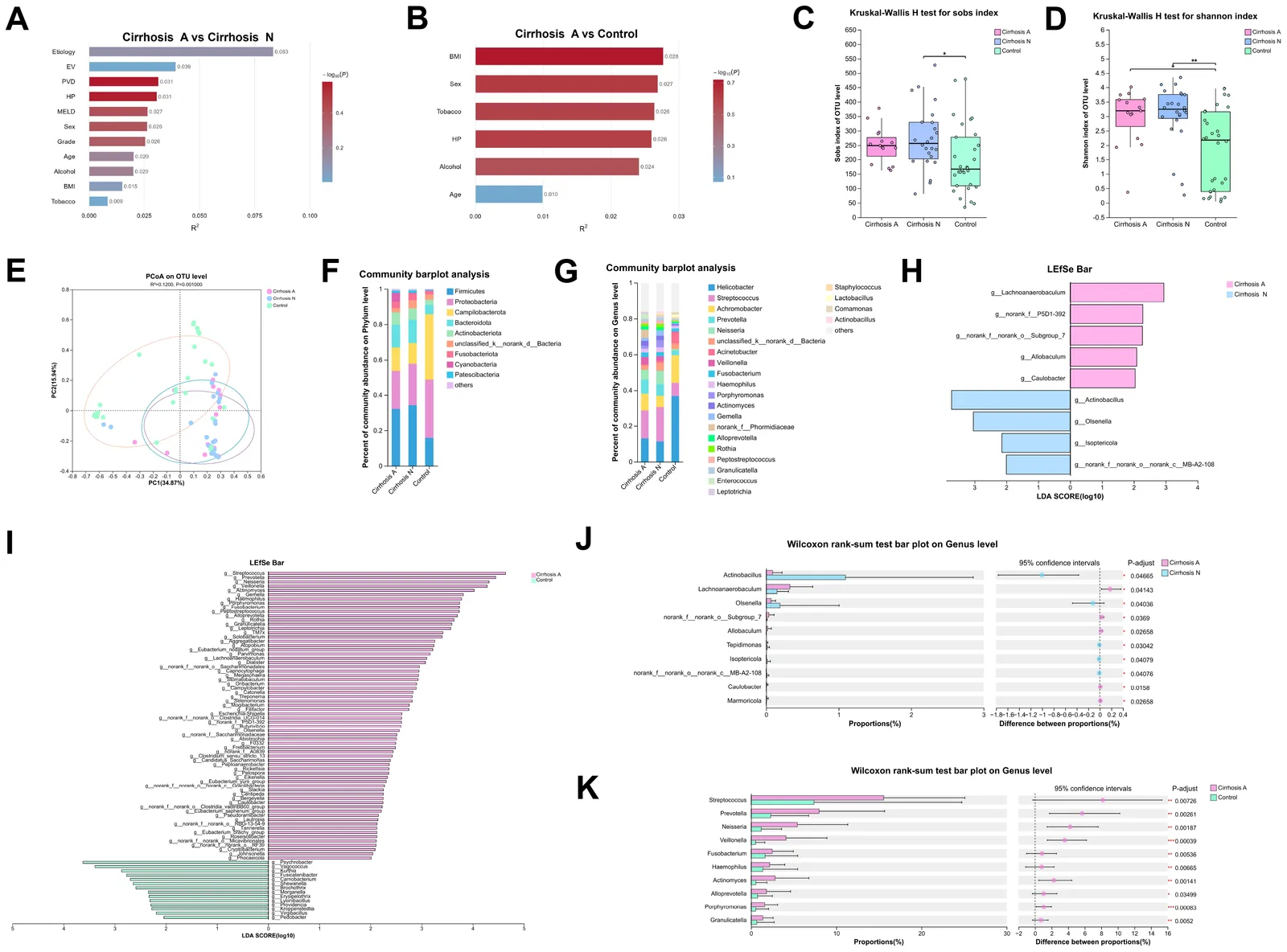
Alterations in gastric microbiota in cirrhosis A compared with cirrhosis N and control. **(A)** PERMANOVA analysis of gastric mucosal microbiota between Cirrhosis A and Cirrhosis N, with liver-related (Child–Pugh, MELD, etiology, varices, PVD) and non-liver-related covariates (age, sex, BMI, smoking, alcohol, and *H. pylori*) included. **(B)** PERMANOVA analysis of gastric mucosal microbiota between Cirrhosis A and Control, with non-liver-related covariates (age, sex, BMI, smoking, alcohol, and *H. pylori*) included. All covariates had P > 0.05. **(C, D)** The Sobs and Shannon indices were used to evaluate the gastric microbial diversity of Cirrhosis A, Cirrhosis N, and Control. **(E)** PCoA of weighted UniFrac distance demonstrated that the three groups showed three distinct clusters. **(F, G)** Relative microbial abundance in the gastric tissue of the three groups at the phylum and genus levels. **(H)** Differential genera between Cirrhosis A and Cirrhosis N identified by LEfSe analysis (LDA > 2.0, Q < 0.05). **(I)** Differential genera between Cirrhosis A and Control identified by LEfSe analysis (LDA > 2.0, Q < 0.05). **(J)** Differential genera between Cirrhosis A and Cirrhosis N identified by the Wilcoxon rank-sum test (adjusted *P*-value < 0.05). **(K)** Differential genera between Cirrhosis A and Control identified by the Wilcoxon rank-sum test (adjusted *P*-value < 0.05). *, q < 0.05; **, q < 0.01; ***, q < 0.001.

For the gastric mucosal microbiota, PERMANOVA analysis similarly showed that all included clinical covariates had no significant effects in either the comparison between Cirrhosis A and Cirrhosis N (**Figure 3A**) or the comparison between Cirrhosis A and Control (**Figure 3B**) (all *P* > 0.05), with *R^2^* values ranging from 0.009 to 0.083, suggesting that these factors did not confound the observed microbial differences. We also measured gastric microbial alpha-diversity using different methodologies. The Sobs and Shannon indices revealed that the microbial community richness and diversity in the gastric mucosa of Cirrhosis A and Cirrhosis N were significantly higher than those of Control (**Figures 3C, D**). Analysis of beta-diversity via PCoA revealed differences in gastric bacterial communities among Cirrhosis A, Cirrhosis N, and Control (*P*-value = 0.001, **Figure 3E**). At the phylum level, the top four pancreatic bacteria identified in the gastric mucosa of Cirrhosis A, Cirrhosis N, and Control were *Firmicutes*, *Proteobacteria*, *Campilobacterota*, and *Bacteroidota*, indicating a broad phylum-level similarity (**Figure 3F**). However, differences emerged at the genus level. The top five genera in the gastric mucosa of Cirrhosis A were *Streptococcus*, *Helicobacter*, *Achromobacter*, *Prevotella*, and *Neisseria*. *Streptococcus*, *Helicobacter*, *Neisseria*, *Achromobacter*, and *Prevotella* were the top five genera detected in the gastric mucosa of Cirrhosis N. The top five genera in the gastric mucosa of Control were *Helicobacter*, *Achromobacter*, *Streptococcus*, *Acinetobacter*, and *unclassified_k:norank_d:Bacteria* (**Figure 3G**). LEfSe (LDA > 2.0, adjusted *P*-value < 0.05) found that *Lachnoanaerobaculum*, *Caulobacter*, and *g:norank_f:P5D1–392* were enriched in the gastric mucosa of Cirrhosis A compared with Cirrhosis N (**Figure 3H**), Control (**Figure 3I**). After *P*-value correction by the BH method, the Wilcoxon rank-sum test (adjusted *P*-value < 0.05) also showed that *Lachnoanaerobaculum*, *Caulobacter*, and *g:norank_f:P5D1–392* were enriched in the gastric mucosa of Cirrhosis A compared with Cirrhosis N (**Figure 3J**; **Supplementary Table 3**); Control (**Figure 3K**, **Supplementary Table 3**).

### Alterations of the gastric transcriptome in cirrhosis A compared with cirrhosis N

**Figure 4:**
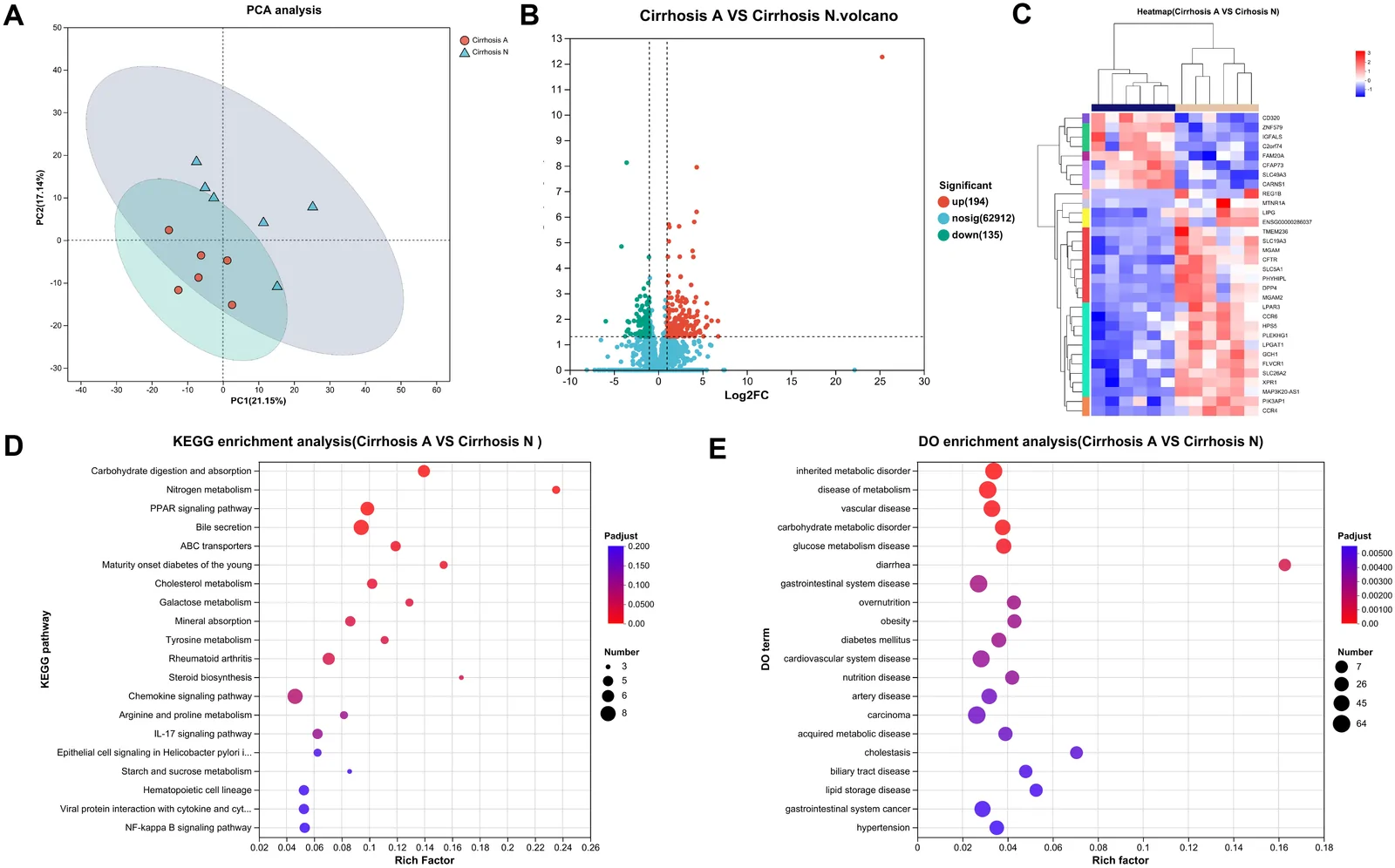
Alterations of the gastric transcriptome in cirrhosis A compared with cirrhosis N. **(A)** PCA plot showing sample distribution between Cirrhosis A and Cirrhosis N along the first two principal components (PC1 and PC2). Dots represent individual samples, colored by group and symbolized by replicate status. **(B)** Volcano plot of differentially expressed genes (DEGs) between Cirrhosis A and Cirrhosis N. Red dots: significantly upregulated genes; green dots: significantly downregulated genes; blue dots: non-significant genes (adjusted *P*-value < 0.05, |log2(fold change)| > 1). **(C)** Heatmap of z-score transformed expression values for 30 clustered genes across 12 samples. Red: high expression; blue: low expression. **(D)** KEGG pathway enrichment analysis for DEGs. Circle size indicates the number of DEGs per pathway; color intensity denotes statistical significance (adjusted P-value), with darker colors indicating more significant pathways. The top 20 enriched pathways are labeled. **(E)** Bubble chart displaying the top 20 enriched diseases among DEGs. X-axis: Rich Factor; Y-axis: diseases. Bubble size reflects the number of associated DEGs; color indicates significance level.

To elucidate the mechanisms underlying gastric mucosal alterations in cirrhosis, we performed transcriptome analysis on six Cirrhosis A and six Cirrhosis N gastric mucosal samples. Principal component analysis (PCA) of the normalized expression data demonstrated distinct transcriptomic clustering patterns for Cirrhosis A samples relative to Cirrhosis N samples (**Figure 4A**). After *P*-value correction by the BH method, 329 genes (adjusted *P*-value < 0.05 and |log2(fold change)| > 1) altered significantly in Cirrhosis A compared with Cirrhosis N, including 194 upregulated genes and 135 downregulated genes (**Figure 4B**, **Supplementary Table 4**). Heatmap showed that *REG1B*, *MGAM2*, *MAP3K20-AS1*, *FLVCR1*, *CFTR*, and other genes were upregulated, while *CARNS1*, *IGFALS*, *ZNF579*, *SLC49A3*, *C2orf74*, and other genes were significantly downregulated (**Figure 4C**). The differentially expressed genes (DEGs) were enriched in multiple KEGG pathways, including the carbohydrate digestion and absorption, nitrogen metabolism, PPAR signaling, bile secretion, ABC transporters, and cholesterol metabolism pathways (adjusted *P*-value < 0.05, **Figure 4D**, **Supplementary Table 5**) Notably, the carbohydrate digestion and absorption pathway was the top enriched pathway. To explore the potential clinical implications of the differential gene expression patterns, we conducted Disease Ontology (DO) enrichment analysis (**Figure 4E**). The top five significantly enriched categories (adjusted *P*-value < 0.05) among the DEGs were inherited metabolic disorder, disease of metabolism, vascular disease, carbohydrate metabolic disorder, and glucose metabolism disease, as detailed in **Supplementary Table 6**. This enrichment analysis provides insights into the potential clinical relevance of the observed transcriptomic changes, highlighting key disease associations that may underlie gastric mucosal alterations in patients with cirrhosis. Furthermore, we mapped the DEGs to GO terms and Reactome pathways (**Supplementary Figures 1, 2**).

### Alterations of the gastric transcriptome in cirrhosis A compared with control A

**Figure 5:**
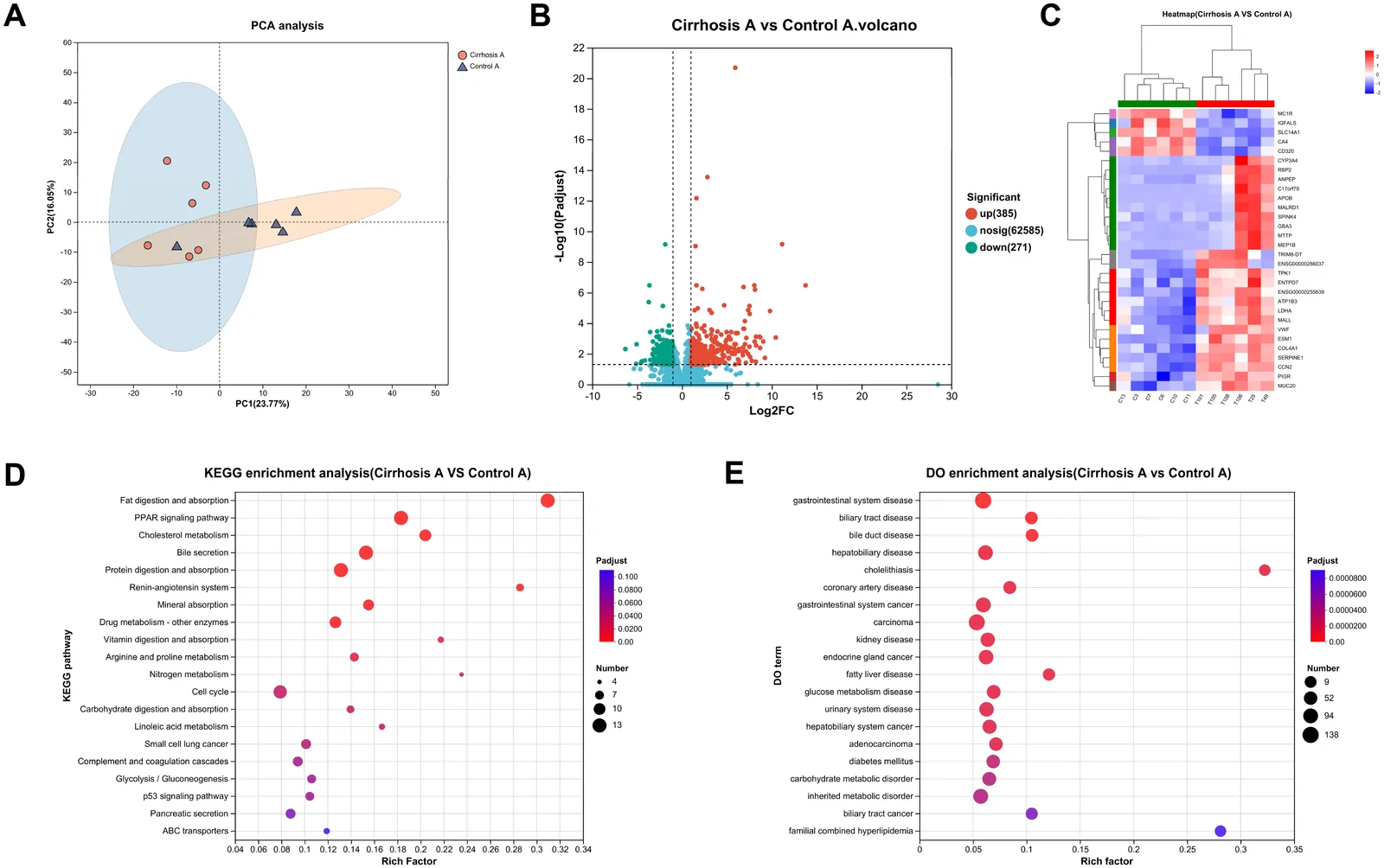
Alterations of the gastric transcriptome in cirrhosis A compared with control A. **(A)** PCA plot showing sample distribution between Cirrhosis A and Control A along the first two principal components (PC1 and PC2). Dots represent individual samples, colored by group and symbolized by replicate status. **(B)** Volcano plot of DEGs between Cirrhosis A and Control A. Red dots: significantly upregulated genes; green dots: significantly downregulated genes; blue dots: non-significant genes (adjusted *P*-value < 0.05, |log2(fold change)| > 1). **(C)** Heatmap of z-score transformed expression values for 30 clustered genes across 12 samples. Red: high expression; blue: low expression. **(D)** KEGG pathway enrichment analysis for DEGs. Circle size indicates the number of DEGs per pathway; color intensity denotes statistical significance (adjusted P-value), with darker colors indicating more significant pathways. The top 20 enriched pathways are labeled. **(E)** Bubble chart displaying the top 20 enriched diseases among DEGs. X-axis: Rich Factor; Y-axis: diseases. Bubble size reflects the number of associated DEGs; color indicates significance level.

We performed transcriptome analysis on six Cirrhosis A and six Control A (CAG patients without LC) gastric mucosal samples. PCA of the normalized expression data demonstrated distinct transcriptomic clustering patterns for Cirrhosis A samples relative to Control A samples (**Figure 5A**). After *P*-value correction by the BH method, 656 genes (adjusted *P*-value < 0.05 and |log2(fold change)| > 1) were significantly altered in Cirrhosis A compared with Control A, including 385 upregulated genes and 271 downregulated genes (**Figure 5B**; **Supplementary Table 7**). Heatmap showed that *SLC51A*, *SLC51B*, *CDX2*, *MUC2*, *APOB*, and other genes were significantly upregulated, while PGA4, *IGKV1D-12*, *MROH2A*, *IGFALS*, and other genes were significantly downregulated (**Figure 5C**). The DEGs were enriched in multiple KEGG pathways, including fat digestion and absorption, PPAR signaling pathway, cholesterol metabolism, bile secretion, protein digestion and absorption, the renin-angiotensin system, and mineral absorption pathways (adjusted *P*-value < 0.05, **Figure 5D**; **Supplementary Table 8**) Among these, the fat digestion and absorption pathway was the most prominently represented pathway. To investigate the potential clinical implications of the differential gene expression patterns, we also performed DO enrichment analysis (**Figure 5E**). The top five significantly enriched categories among the DEGs were gastrointestinal system disease, biliary tract disease, bile duct disease, hepatobiliary disease, and cholelithiasis (adjusted *P*-value < 0.05), as detailed in **Supplementary Table 9**. Furthermore, we mapped the DEGs to GO terms and Reactome pathways (**Supplementary Figures 1, 2**).

### Alterations of the gastric transcriptome in cirrhosis A compared with cirrhosis N and control A

**Figure 6:**
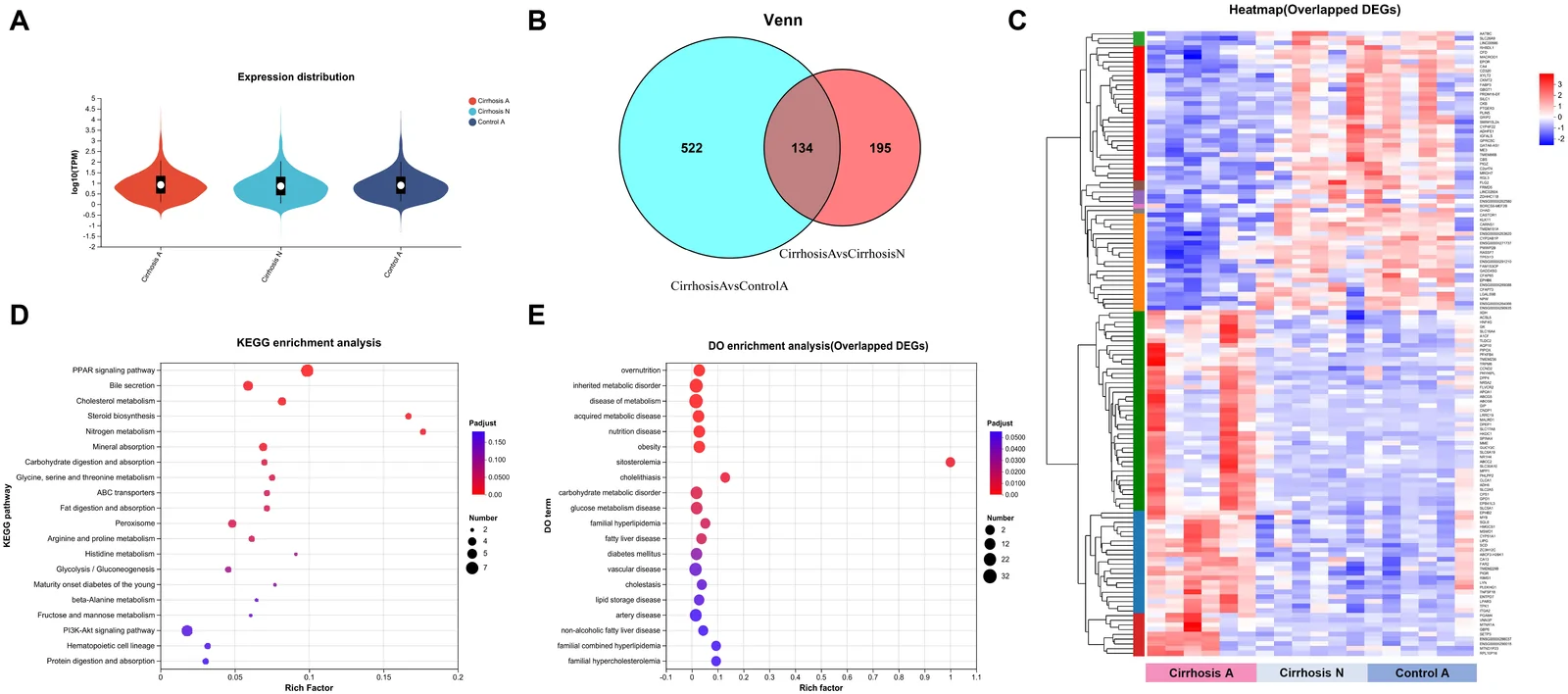
Alterations of the gastric transcriptome in cirrhosis A compared with cirrhosis N and control A. **(A)** Violin plot showing log2-normalized gene expression distributions. Boxes denote interquartile ranges, lines represent medians, and outliers are shown as individual points. **(B)** Venn diagram illustrating the overlap of DEGs in the gastric mucosa of Cirrhosis A compared with Cirrhosis N and Control A. The intersection indicates overlapping DEGs, and non-overlapping regions show unique DEGs. **(C)** Heatmap of z-score transformed expression values for 30 overlapping DEGs. Red: high expression; blue: low expression. **(D)** KEGG pathway enrichment analysis for overlapping DEGs. Circle size indicates the number of DEGs per pathway; color intensity denotes statistical significance (adjusted P-value), with darker colors indicating more significant pathways. The top 20 enriched pathways are labeled. **(E)** Bubble chart displaying the top 20 enriched diseases among overlapping DEGs. X-axis: Rich Factor; Y-axis: diseases. Bubble size reflects the number of associated overlapping DEGs; color indicates significance level.

The violin plot showed that the median lines for each sample remained at a similar level, indicating comparable expression levels across samples within the three groups (**Figure 6A**). Venn diagrams illustrated the number of DEGs between Cirrhosis A and Cirrhosis N (329, 2.16%) was smaller than the number between Cirrhosis A and Control A (656, 4.24%, **Figure 6B**), which suggested that LC progression may result in more pronounced molecular alterations in the gastric mucosa. Some of the DEGs in Cirrhosis A, compared with Cirrhosis N and Control A, were the same, as were the trends of content changes in Cirrhosis A. For example, *ABCG5*, *ITGA2*, *LPAR3*, *PHLPP2*, *MYB*, and other genes were upregulated, whereas EPOR, *CHAD*, *CARNS1*, *IGFALS*, *CFAP73*, and other genes were downregulated in Cirrhosis A compared with both Cirrhosis N and Control A (adjusted *P*-value < 0.05, **Figure 6C**). These overlapping DEGs were enriched in multiple KEGG pathways, including PPAR signaling, bile secretion, cholesterol metabolism, steroid biosynthesis, nitrogen metabolism, mineral absorption, and PI3K-Akt signaling pathways (**Figure 6D**). Furthermore, DO analysis of these overlapping DEGs identified associations with several diseases, including overnutrition, inherited metabolic disorder, and disease of metabolism (**Figure 6E**). These findings provide insights into the molecular mechanisms underlying gastric mucosal changes in patients with LC, highlighting key pathways involved in their pathogenesis and their potential clinical implications.

### Alterations in the microbiota and transcriptome in control A compared with control N

To further evaluate the impact of CAG in the absence of cirrhosis, we compared Control A and Control N (healthy controls without LC or CAG). PCoA analysis revealed no significant separation between the two groups in either salivary microbiota (*P* = 0.437, **Supplementary Figure 3A**) or gastric mucosal microbiota (*P* = 0.102, **Supplementary Figure 3B**), suggesting that CAG alone, without concurrent LC, did not substantially alter the microbial community structure. LEfSe analysis identified several differential genera between Control A and Control N in both saliva and gastric mucosa (LDA > 2.0, adjusted *P*-value < 0.05, **Supplementary Figures 3B, D**). However, PCA analysis of the gastric mucosal transcriptome showed no distinct clustering pattern between the two groups (**Supplementary Figure 3E**), and after BH multiple-testing correction, only three genes met the significance threshold of adjusted *P*-value < 0.05 and |log2FC| > 1: *IGKV1D-17* and *HLA-L* were upregulated, while *FOLR1* was downregulated in Control A compared with Control N (**Supplementary Figure 3F**). Collectively, these findings suggest that, in the absence of LC, CAG alone may not drive substantial alterations in the microbiota or transcriptome, further supporting the hypothesis that LC plays a dominant role in shaping the observed microbial and molecular changes in patients with LC and CAG.

### Correlation network between salivary differential genera and gastric mucosal gene expression

**Figure 7:**
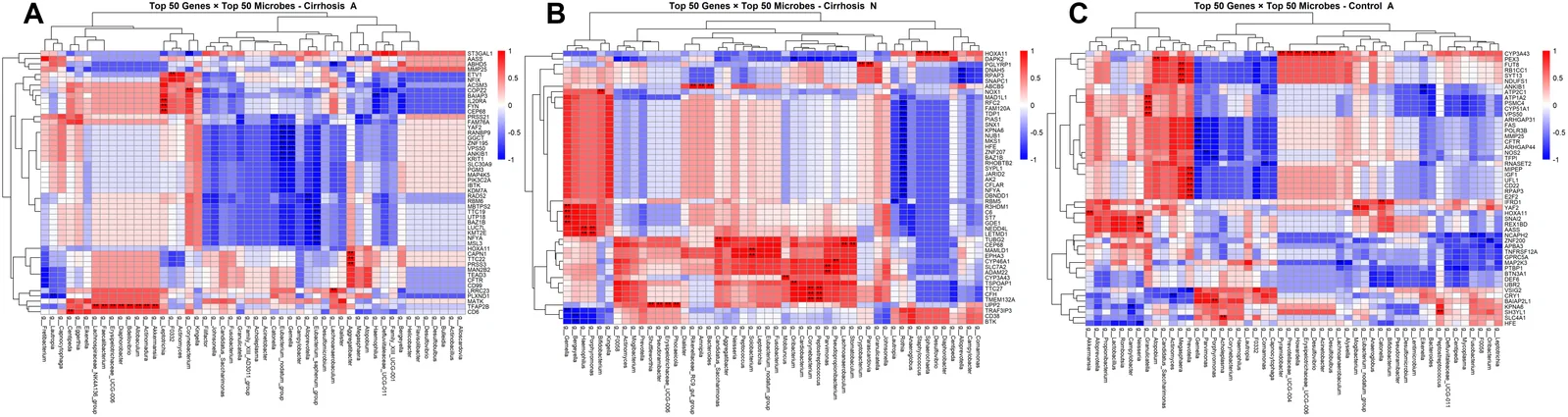
The correlation analysis between top 50 differential genera and gastric mucosal gene expression identified by Spearman's correlation method. Correlation heatmap in the Cirrhosis A **(A)**, Cirrhosis N **(B)**, and Control A **(C)** groups. Rows represent DEGs; columns represent differential genera. Red indicates positive correlation; blue indicates negative correlation. **p < 0.05.

Spearman correlation analysis revealed significant associations between the top 50 differential genera and DEGs in the gastric mucosa. In the Cirrhosis A group, increased *Aggregatibacter* abundance was positively correlated with the *CAPN1 and* the cancer-associated genes *TTC22* and *PRSS3* (**Figure 7A**). Increased *Leptotrichia* was positively correlated with *FYN*, CEP68, and the pro-inflammatory signaling receptor *IL20RA*. Increased *Allobaculum* was positively correlated with the procarcinogenic transcription factor *TFAP2B*. Increased *Centipeda* was positively correlated with the T-cell marker *CD6*. Increased *Eubacterium_saphenum_group* was negatively correlated with nucleolar function-related genes, including *MBTPS2*, *UTP18*, and *BAZ1B*. In the Cirrhosis N group, *Gemella* was positively correlated with *R3HDM1* and *GDE1* (**Figure 7B**). In the Control group, decreased *Prevotella* was positively correlated with *MIPEP*, *IGF1*, and *UFL1* (**Figure 7C**).

## Discussion

To the best of our knowledge, this is the first study to explore the alterations in the microbiota and transcriptome in the saliva and gastric mucosa of patients with CAG and LC. Notably, DEGs in the gastric mucosa of Cirrhosis A were significantly enriched in the bile secretion and cholesterol metabolism pathways, accompanied by elevated expression of the intestinal markers *CDX2* and *MUC2*, which may be associated with the development of GIM or CAG. These findings suggest a possible link between dysregulation of bile secretion-related pathways and gastric mucosal changes in patients with LC. Furthermore, we successfully developed non-invasive diagnostic models with high accuracy using the shared differential salivary genera in Cirrhosis A compared with Cirrhosis N and Control.

Our study confirmed a shared presence and reduction of certain genera in Cirrhosis A compared with Cirrhosis N and Control. *Veillonella* was enriched in the saliva of Cirrhosis A compared with both Cirrhosis N and Control. *Veillonella* was observed to have higher relative abundance in patients with GC compared to those with gastritis and healthy controls and has been identified as a potential biomarker for GC (Nath and Natarajan, 2024). Whether the microbe is involved in the occurrence of CAG associated with LC requires further investigation.

In our study, we observed the dysfunction of the PI3K-Akt signaling pathway in the gastric mucosa of Cirrhosis A, with upregulation of *ITGA2*, *LPAR3*, *PHLPP2*, and *MYB* and downregulation of *EPOR* and *CHAD*. The PI3K/Akt signaling pathway is constitutively activated in various cancers, including breast cancer, GC, and pancreatic cancer (Amin et al., 2015; Hao et al., 2015). *ITGA2* is an important molecule involved in tumor cell proliferation, survival, and migration, and its upregulation may reflect structural changes in the gastric mucosa and activation of epithelial-to-mesenchymal transition (EMT) (San Antonio et al., 2009). *LPAR3* and *MYB* are also involved in tumor cell proliferation and migration (Brusevold et al., 2014; Li et al., 2016), and may drive the development of precancerous lesions of GC (PLGC). Whether LC is associated with the occurrence of CAG and its potential further progression toward GC via the PI3K-Akt signaling pathway warrants further investigation.

DEGs in the gastric mucosa of Cirrhosis A were also significantly enriched in bile secretion, cholesterol metabolism, and PPAR signaling pathways. In our study, *ABCG5* and *ABCG8* were significantly upregulated in the gastric mucosa of Cirrhosis A compared with both Cirrhosis N and Control A and have been shown to play important roles in regulating bile secretion and sterol content in bile (Klett et al., 2004). We also found that *SLC51A* and *SLC51B*, which encode the organic solute transporter alpha-beta (Ostα–Ostβ), were upregulated. Ostα–Ostβ is responsible for transporting bile acids (BAs) across the enterocyte basolateral membrane into the portal circulation (Dawson et al., 2005). Upregulation of these genes may represent an adaptive change in gastric mucosal cells in response to bile reflux. Previous studies have shown that bile reflux could promote the development of PLGC, including CAG or GIM, by activating the IL-6/Janus kinase 1/*STAT3* signaling pathway (Wang et al., 2022). Yu et al. found that BAs could promote GIM by upregulating *CDX2* and *MUC2* expression via the FXR/NF-κB signaling pathway (Yu et al., 2019). In our study, *CDX2* and *MUC2* were also significantly upregulated in the gastric mucosa of Cirrhosis A compared with Control A, suggesting that LC may be associated with the expression of intestinal markers such as *CDX2* and *MUC2* in the gastric mucosa through bile reflux into the stomach, which may be associated with the development of GIM or CAG (Li et al., 2019). Furthermore, the PPAR signaling pathway plays a crucial role in regulating lipid metabolism and maintaining hepatic homeostasis, and its dysregulation is linked to liver fibrosis, a precursor to LC (Pawlak et al., 2015; Guo et al., 2024). Dysregulation of this pathway is also linked to CAG. PPAR-γ exerts anti-inflammatory and immunomodulatory effects by decreasing NF-κB p65 activity, thereby alleviating inflammation and slowing the progression of glandular atrophy and fibrosis (Cuzzocrea, 2004).

Additionally, the DO analysis highlights several disease categories enriched in Cirrhosis A, including metabolic diseases, carcinoma, and gastrointestinal system disorders. These findings align with the understanding that cirrhosis often coexists with metabolic dysfunction and increases the risk of gastrointestinal malignancies (Schuppan and Afdhal, 2008). These results emphasize the importance of the aforementioned molecular pathways in LC progression and may inform future therapeutic strategies aimed at mitigating CAG associated with LC.

In this study, Spearman correlation analysis identified significant associations between salivary differential genera and key gastric mucosal genes. In patients with LC-associated CAG, the positive correlation between increased *Aggregatibacter* and the procarcinogenic genes *PRSS3* warrants attention. *Aggregatibacter actinomycetemcomitans* has been reported to be significantly associated with the risk of PLGC, and its elevation is an important predictor of increased risk of precancerous gastric lesions (Gao et al., 2025). *PRSS3* is significantly overexpressed in GC tissues, and its high expression is significantly correlated with postoperative metastasis and poor prognosis (Pang et al., 2026). The positive correlation between increased *Allobaculum* and the procarcinogenic transcription factor *TFAP2B* suggests that specific microbiota may participate in GC progression through transcriptional regulation. *TFAP2B* can be recruited by exosomal circPTBP3 to target gene promoters in GC, promoting peritoneal metastasis (Dong et al., 2025). Causal relationships require validation through longitudinal cohort studies and functional experiments.

Our study has several limitations. First, the relatively small sample size may limit the statistical power and generalizability of our findings, and external validation of the diagnostic model in independent multicenter cohorts with larger samples is still needed to further confirm the robustness of our conclusions. Second, the lack of an independent external validation cohort restricts model generalizability, and although five-fold cross-validation was used to minimize overfitting, external validation remains essential for clinical applicability. Third, due to the observational nature of the study, it remains unclear whether the identified microbial and transcriptomic alterations are causes or consequences of CAG in the context of LC. Additional functional studies, both *in vivo* and *in vitro*, are essential to elucidate the potential causal mechanisms. Fourth, the transcriptomic analysis was performed on a limited number of samples, which may restrict statistical power and increase the risk of false-positive findings. Although we applied rigorous BH multiple-testing correction (adjusted *P*-value < 0.05) to minimize false discoveries, we acknowledge that these findings remain exploratory, and key DEGs require further validation by qPCR in independent cohorts.

To the best of our knowledge, this study is the first to develop robust models for distinguishing Cirrhosis A from Cirrhosis N and Controls, while simultaneously characterizing the microbial and transcriptomic landscapes in the saliva and gastric mucosa of patients with LC and CAG. Future investigations exploring the precise functions of these differentially abundant microbes, DEGs, and enriched pathways may provide deeper insights into their clinical significance and potential therapeutic targets.

## Data Availability

The datasets presented in this study can be found in online repositories. The names of the repository/repositories and accession number(s) can be found below: https://figshare.com/, 10.6084/m9.figshare.27217092, https://figshare.com/, 10.6084/m9.figshare.27217107, https://figshare.com/, 10.6084/m9.figshare.27216927, https://figshare.com/, 10.6084/m9.figshare.27216741, https://figshare.com/, 10.6084/m9.figshare.27216984, https://figshare.com/, 10.6084/m9.figshare.27217059, https://figshare.com/, 10.6084/m9.figshare.27217221.

## Glossary

ABCG: ATP binding cassette subfamily G member
APOB: apolipoprotein B
AUC: area under the curve
BA: bile acid
BH: Benjamini–Hochberg
CAG: chronic atrophic gastritis
Cirrhosis A: liver cirrhosis patients with chronic atrophic gastritis
Control: controls without LC
Control A: CAG patients without LC
CARNS1: carnosine synthase
CDX2: caudal type homeobox 2
CFAP73: centrosomal filament-associated protein 73
CFTR: CF transmembrane conductance regulator
CHAD: chondroadherin
Cirrhosis N: liver cirrhosis patients without chronic atrophic gastritis
C2orf74: chromosome 2 open reading frame 74
DEG: differentially expressed gene
DO: Disease Ontology
EPOR: erythropoietin receptor
FDR: false discovery rate
FLVCR1: FLVCR heme transporter 1
GC: gastric cancer
GO: Gene Ontology
GIM: gastric intestinal metaplasia
HCC: hepatocellular carcinoma
IBD: inflammatory bowel disease
IGFALS: insulin-like growth factor binding protein acid labile subunit
IGKV1D-12: immunoglobulin kappa variable 1D-12
IL: interleukin
ITGA2: integrin subunit alpha 2
KEGG: Kyoto Encyclopedia of Genes and Genomes
LC: liver cirrhosis
LDA: linear discriminant analysis
LEfSe: linear discriminant analysis effect size
LPAR3: lysophosphatidic acid receptor 3
MAP3K20-AS1: MAP3K20 antisense RNA 1
MELD: model for end-stage liver disease
MGAM2: maltase-glucoamylase 2
MROH2A: maestro heat like repeat family member 2A
MUC: mucin
MYB: MYB proto-oncogene, transcription factor
Ostα–Ostβ: organic solute transporter alpha-beta
OTU: operational taxonomic unit
PCA: principal component analysis
PCoA: principal coordinate analysis
PCR: polymerase chain reaction
PGA4: pepsinogen A4
PHLPP2: PH domain and leucine rich repeat protein phosphatase 2
PLGC: precancerous lesions of gastric cancer
PPAR: peroxisome proliferator-activated receptor
PPI: proton-pump inhibitor
PVD: portal vein diameter
REG1B: regenerating family member 1 beta
RF: random forest
ROC: receiver operating characteristic curve
ROS: reactive oxygen species
rRNA: ribosomal RNA
RUT: rapid urease test
SLC: solute carrier family
STAT3: signal transducer and activator of transcription 3
ZNF579: zinc finger protein 579
