# Correction: Integrated microbial and transcriptomic profiling reveals distinct correlations in atrophic gastritis associated with liver cirrhosis

**DOI:** 10.3389/fcimb.2026.1979008

**Published:** 2026-09-09

**Authors:** Yiqing Zhu, Guoming Zhang, Ruiguang Ma, Qian Li, Huayu Dai, Zhouyue Li, Lixiang Li, Zhen Li

**Affiliations:** 1 Department of Gastroenterology, Qilu Hospital of Shandong University, Jinan, Shandong, China; 2 Shandong Provincial Clinical Research Center for Digestive Disease, Jinan, Shandong, China; 3 Laboratory of Translational Gastroenterology, Qilu Hospital of Shandong University, Jinan, Shandong, China; 4 Robot Engineering Laboratory for Precise Diagnosis and Therapy of GI Tumor, Qilu Hospital of Shandong University, Jinan, Shandong, China; 5 Department of Gastroenterology, The Affiliated Hospital of Qingdao University, Qingdao, Shandong, China; 6 Department of Gastroenterology, Binzhou Medical University Hospital, Binzhou, Shandong, China

**Keywords:** chronic atrophic gastritis, liver cirrhosis, machine learning, microbiota, transcriptome

An incorrect number was provided for the “ECCM Program of Clinical Research Center of Shandong University”. The correct number is “2021SDUCRCB004”.

The original version of this article has been updated.

