# Supplementary material for: Integrated microbial and transcriptomic profiling reveals distinct correlations in atrophic gastritis associated with liver cirrhosis
Source: Front Cell Infect Microbiol. 2026 Aug 21;16:1893991. doi: 10.3389/fcimb.2026.1893991 (PMC13541563; doi:10.3389/fcimb.2026.1893991)
Supplement: Supplementary file 1 [file DataSheet1.pdf]

SUPPLEMENTARY FIGURES

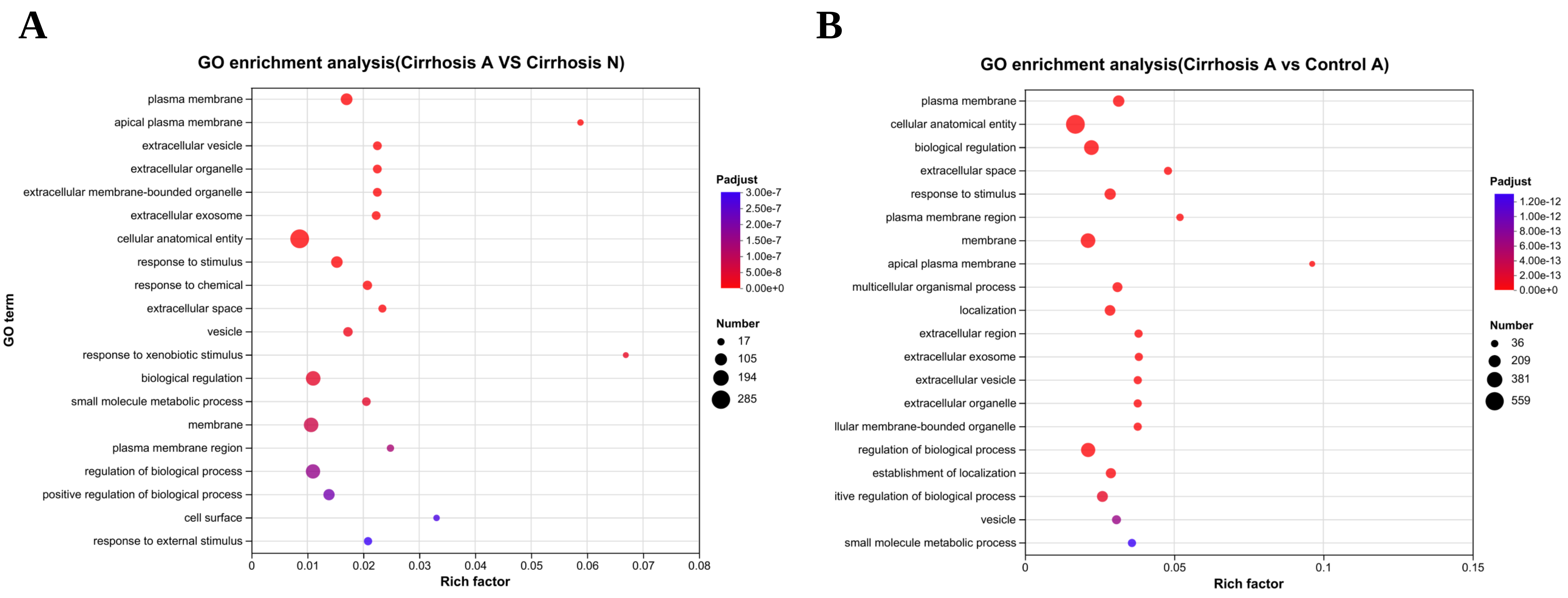

Fig S1 Top 20 GO enrichment terms in Cirrhosis A compared to Cirrhosis N (A) and Control A (B) in the gastric mucosa. Bubble sizes reflect the number of associated genes, and colors indicate statistical significance (P-adjust).

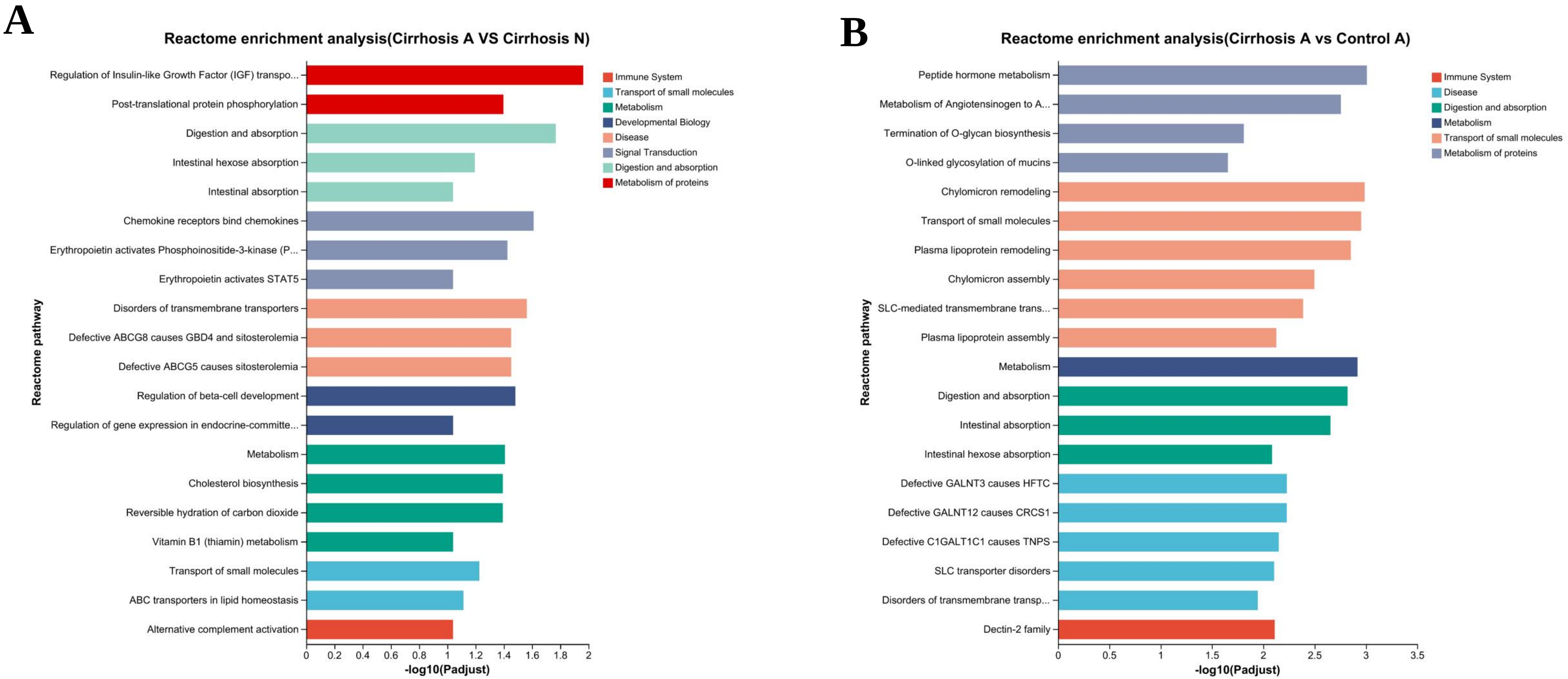

Fig S2 Reactome pathways in Cirrhosis A compared to Cirrhosis N (A) and Control A (B) in the gastric mucosa. Bar plot showing top enriched Reactome pathways. Bars represent the number of annotated genes, The colors represent disease classifications and X-axis indicate statistical significance (−log10(P-adjust)).

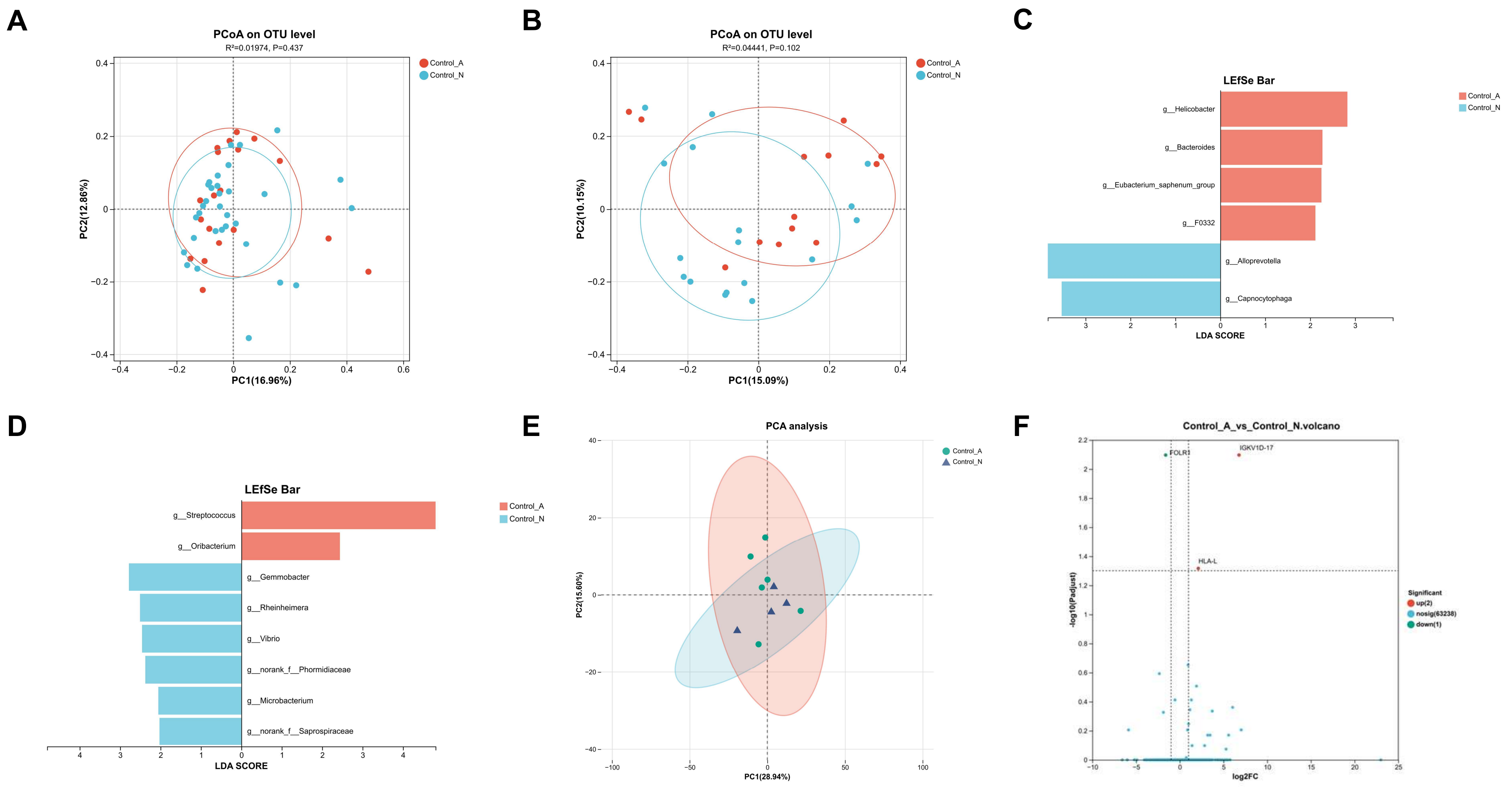

Fig S3 Comparisons between Control A and Control. A PCoA analysis of salivary microbiota. **B** LEfSe analysis of differential salivary genera (LDA > 2.0, Q < 0.05). **C** PCoA analysis of gastric mucosal microbiota. **D** LEfSe analysis of differential gastric mucosal genera (LDA > 2.0, Q < 0.05). **E** PCA analysis of gastric mucosal transcriptome. **F** Volcano plot of differentially expressed genes (adjusted P-value < 0.05, |log2FC| > 1).
