# Supplementary material for: Integrated microbial and transcriptomic profiling reveals distinct correlations in atrophic gastritis associated with liver cirrhosis
Source: Front Cell Infect Microbiol. 2026 Aug 21;16:1893991. doi: 10.3389/fcimb.2026.1893991 (PMC13541563; doi:10.3389/fcimb.2026.1893991)
Supplement: Supplementary file 2 [file Table1.docx]

**Integrated Microbial and Transcriptomic Profiling Reveals Distinct Correlations in Atrophic Gastritis Associated with Liver Cirrhosis**

**Supplementary Appendix**

**Yiqing Zhu^1,2,3,4^†, Guoming Zhang^1,2,3,4^†,** Ruiguang Ma^5^, Qian Li^6^, Huayu Dai^1,2,3,4^, Zhouyue Li^1,2,3,4^, Lixiang Li^1,2,3,4^, Zhen Li^1,2,3,4^*

^1^Department of Gastroenterology, Qilu Hospital of Shandong University, Jinan, Shandong, China

^2^Shandong Provincial Clinical Research Center for Digestive Disease, Shandong, China

^3^Laboratory of Translational Gastroenterology, Qilu Hospital of Shandong University, Jinan, Shandong, China

^4^Robot engineering laboratory for precise diagnosis and therapy of GI tumor, Qilu Hospital of Shandong University, Jinan, Shandong, China

^5^Department of Gastroenterology, The Affiliated Hospital of Qingdao University, Qingdao, Shandong, China

^6^Department of Gastroenterology, Binzhou Medical University Hospital, Binzhou, Shandong, China

† These authors have contributed equally to this work and share first authorship.

* Correspondence author: Zhen Li

**Contents**

**SUPPLEMENTARY TABLES** ..………...………...……………………………….. 3

**Table S1** Clinicopathological characteristics of patients in this study ..................... 3

**Table S2** Different microorganisms in the saliva of Cirrhosis A compared to Cirrhosis N and Control respectively ........................................................................... 5

**Table S3** Different microorganisms in the gastric mucosa of Cirrhosis A compared to Cirrhosis N and Control respectively ……...……………………………………… 6

**Table S4** Top 100 DEGs between Cirrhosis A and Cirrhosis N in the gastric mucosa ………………………………………………………………………………..8

**Table S5** KEGG enrichment pathways of DEGs between Cirrhosis A and Cirrhosis N in the gastric mucosa............................................................................................... 10

**Table S6** DO enrichment of DEGs between Cirrhosis A and Cirrhosis N in the gastric mucosa ............................................................................................................ 11

**Table S7** Top 100 DEGs between Cirrhosis A and Control A in the gastric mucosa..........................................................................................................................13

**Table S8** KEGG enrichment pathways of DEGs between Cirrhosis A and Control A in the gastric mucosa ……...……………………………...……………..……….…. 15

**Table S9** DO enrichment of DEGs between Cirrhosis A and Control A in the gastric mucosa …………………………………………………………...…………………. 16

**Table S10** Summary of sequencing quality control metrics for 16S rRNA amplicon sequencing…………………………………...…………………...…………………. 19

**Table S1 Clinicopathological characteristics of patients in this study**

| **Omics type** | **Characteristic** | **Cirrhosis A** | **Cirrhosis N** | **Control** |
| --- | --- | --- | --- | --- |
| **Oral microbiota genomic** | **Number** | 25 | 48 | 51 |
|  | **Gender** |  |  |  |
|  | Male | 9 | 24 | 26 |
|  | Female | 16 | 24 | 25 |
|  | ***P*-Value** |  | **Cirrhosis A vs Cirrhosis N** | **Cirrhosis A vs Control** |
|  |  |  | 0.2602 | 0.2896 |
|  | **Age，mean±SD (ys)** | 54.68 ± 13.49 | 53.31 ± 11.75 | 48.35 ± 14.88 |
|  | ***P*-Value** |  | 0.5218 | 0.06609 |
|  | **BMI，mean±SD (Kg/m^2^)** | 23.72 ± 3.59 | 23.96 ± 3.84 | 23.07 ± 3.76 |
|  | ***P*-Value** |  | 0.92 | 0.2434 |
|  | **MELD** | 9.04 ± 2.11 | 9.31 ± 2.26 |  |
|  | ***P*-Value** |  | 0.6118 |  |
|  | **Portal vein diameter** | 1.39 ± 0.21 | 1.40 ± 0.18 |  |
|  | ***P*-Value** |  | 0.8119 |  |
|  | **Alcohol, Yes/No** | 13/12 | 22/26 | 21/30 |
|  | ***P*-Value** |  | 0.5182 | 0.7998 |
|  | **Tobacco, Yes/No** | 10/15 | 17/31 | 24/27 |
|  | ***P*-Value** |  | 0.8970 | 0.7369 |
|  | **HP, Yes/No** | 4/21 | 11/37 | 10/41 |
|  | ***P*-Value** |  | 0.6974 | 0.9999 |
|  | **Esophagogastric varices**  **, 0/1/2/3** | 0/6/6/13 | 2/9/17/20 |  |
|  | ***P*-Value** |  | 0.5747 |  |
|  | **Child-pugh, A/B/C** | 20/4/1 | 29/16/3 |  |
|  | ***P*-Value** |  | 0.2739 |  |
|  | **Etiology (Virus: 1, Alcohol: 2, Autoimmune: 3, Drugs: 4, Others: 5)** | 11/1/5/0/8 | 20/3/7/0/18 |  |
|  | ***P*-Value** |  | 0.9230 |  |
| **Gastric microbiota genomic** | **Number** | 15 | 24 | 30 |
|  | **Gender** |  |  |  |
|  | Male | 12 | 12 | 15 |
|  | Female | 3 | 12 | 15 |
|  | ***P*-Value** |  | 0.06692 | 0.05735 |
|  | **Age，mean±SD (ys)** | 62.2 ± 12.33 | 54 ± 8.854 | 51.366± 11.58 |
|  | ***P*-Value** |  | 0.07469 | 0.05293 |
|  | **BMI，mean±SD (Kg/m^2^)** | 26.0093 ± 3.545 | 25.5208 ± 3.457 | 22.4317 ± 3.4656 |
|  | ***P*-Value** |  | 0.6245 | 0.2477 |
|  | **MELD** | 8.67 ± 2.23 | 9.71 ± 2.54 |  |
|  | ***P*-Value** |  | 0.1879 |  |
|  | **Portal vein diameter** | 0.67 ± 0.49 | 0.46 ± 0.51 |  |
|  | ***P*-Value** |  | 0.2116 |  |
|  | **Alcohol, Yes/No** | 11/4 | 15/19 | 18/12 |
|  | ***P*-Value** |  | 0.6448 | 0.5820 |
|  | **Tobacco, Yes/No** | 8/7 | 8/16 | 15/15 |
|  | ***P*-Value** |  | 0.3677 | 0.9999 |
|  | **HP, Yes/No** | 4/21 | 15/9 | 13/17 |
|  | ***P*-Value** |  | 0.8168 | 0.7513 |
|  | **Esophagogastric varices**  **, 0/1/2/3** | 0/2/3/10 | 1/2/9/12 |  |
|  | ***P*-Value** |  | 0.5787 |  |
|  | **Child-pugh, A/B/C** | 12/3/0 | 13/11/0 |  |
|  | ***P*-Value** |  | 0.1960 |  |
|  | **Etiology (Virus: 1, Alcohol: 2, Autoimmune: 3, Drugs: 4, Others: 5)** | 9/3/3/0/0 | 14/0/5/1/4 |  |
|  | ***P*-Value** |  | 0.0999 |  |
| **Gastric mucosa transcriptomic** | **Numbe**r | 6 | 6 | 6 |
|  | **Gender** |  |  |  |
|  | Male | 5 | 5 | 2 |
|  | Female | 1 | 1 | 4 |
|  | ***P*-Value** |  | 1 | 0.1122 |
|  | **Age，mean±SD (ys)** | 62.5 ± 11.64 | 57.17 ± 12.25 | 61.33 ± 110.37 |
|  | ***P*-Value** |  | 0.5144 | 1 |
|  | **BMI，mean±SD (Kg/m^2^)** | 22.695 ± 3.942 | 26.223 ± 2.818 | 24.285 ± 2.289 |
|  | ***P*-Value** |  | 0.1093 | 0.4273 |
|  | **MELD** | 6.83 ± 2.93 | 8.83 ± 6.79 |  |
|  | ***P*-Value** |  | 0.9999 |  |
|  | **Portal vein diameter** | 1.18 ± 0.17 | 1.18 ± 0.26 |  |
|  | ***P*-Value** |  | 0.9999 |  |
|  | **Alcohol, Yes/No** | 3/3 | 5/1 | 2/4 |
|  | ***P*-Value** |  | 0.5454 | 0.9999 |
|  | **Tobacco, Yes/No** | 2/4 | 4/2 | 1/5 |
|  | ***P*-Value** |  | 0.9999 | 0.9999 |
|  | **HP, Yes/No** | 3/3 | 2/4 | 3/3 |
|  | ***P*-Value** |  | 0.9999 | 0.9999 |
|  | **Esophagogastric varices**  **, 0/1/2/3** | 0/1/3/2 | 0/4/2/10 |  |
|  | ***P*-Value** |  | 0.3563 |  |
|  | **Child-pugh, A/B/C** | 2/4/0 | 3/1/2 |  |
|  | ***P*-Value** |  | 0.5382 |  |
|  | **Etiology (Virus: 1, Alcohol: 2, Autoimmune: 3, Drugs: 4, Others: 5)** | 5/0/1/0/0 | 2/3/0/0/1 |  |
|  | ***P*-Value** |  | 0.1104 |  |

The distribution of gender, age, and BMI in Cirrhosis A, Cirrhosis N, and Control groups. The differences between Cirrhosis N and Control groups compared to Cirrhosis A were assessed using independent samples t-tests or Mann-Whitney U tests.

**Table S2 Different microorganisms in the saliva of Cirrhosis A compared to Cirrhosis N and Control respectively**

| **Microorganisms** | ***P*-adjust** | |
| --- | --- | --- |
|  | **Cirrhosis A vs Cirrhosis N** | **Cirrhosis A vs Control** |
| **Genus** | | |
| *g__Veillonella** | 0.00231 | 0.01038 |
| *g__Atopobium** | 0.03411 | 0.02700 |
| *g__norank_f__P5D1-392** | 0.00444 | 0.00024 |
| *g__Lactobacillus* | 0.04502 | 0.02926 |
| *g__unclassified_f__Moraxellaceae** | 0.04657 | 0.04048 |
| *g__Simonsiella* | 0.04872 | 0.00357 |
| *g__Megasphaera** | 0.0063 | NS |
| *g__Oribacterium** | 0.01934 | NS |
| *g__Stomatobaculum* | 0.02195 | NS |
| *g__Eubacterium_nodatum_group** | 0.01988 | NS |
| *g__Selenomonas** | 0.01175 | NS |
| *g__unclassified_f__Veillonellaceae* | 0.00867 | NS |
| *g__Propionibacterium* | 0.03349 | NS |
| *g__unclassified_f__Saccharimonadaceae* | 0.02592 | NS |
| *g__unclassified_p__Firmicutes* | 0.01331 | NS |
| *g__Mycobacterium** | 0.04653 | NS |
| *g__Actinomyces** | NS | 0.02774 |
| *g__Haemophilus** | NS | 0.02394 |
| *g__Fusobacterium* | NS | 0.03055 |
| *g__Porphyromonas** | NS | 0.04644 |
| *g__Leptotrichia** | NS | 0.02774 |
| *g__Alloprevotella** | NS | 0.03868 |
| *g__Achromobacter** | NS | 0.00074 |
| *g__Gemella** | NS | 0.02983 |
| *g__Capnocytophaga** | NS | 0.00808 |
| *g__norank_f__norank_o__Absconditabacteriales_SR1** | NS | 0.00066 |
| *g__Solobacterium** | NS | 0.04855 |
| *g__Parvimonas* | NS | 0.02102 |
| *g__Campylobacter** | NS | 0.01094 |
| *g__Lautropia** | NS | 0.00598 |
| *g__Sphingomonas** | NS | 0.00056 |
| *g__unclassified_c__Actinobacteria** | NS | 0.04848 |
| *g__Olsenella** | NS | 0.00246 |
| *g__Mycoplasma** | NS | 0.03903 |
| *g__unclassified_f__Enterobacteriaceae* | NS | 0.02375 |
| *g__Scardovia** | NS | 0.00613 |
| *g__Burkholderia-Caballeronia-Paraburkholderia* | NS | 0.01240 |
| *g__unclassified_c__Bacilli* | NS | 0.02723 |
| *g__unclassified_f__Peptostreptococcaceae* | NS | 0.0417 |
| *g__unclassified_f__Actinomycetaceae** | NS | 0.02651 |
| *g__Lentimicrobium* | NS | 0.04369 |
| *g__W5053* | NS | 0.03872 |
| *g__Pseudoramibacter* | NS | 0.01534 |

Red filling: indicates that the microorganism was significantly up-regulated in Cirrhosis A; Blue filling: indicates that the microorganism was significantly down-regulated in Cirrhosis A; Stars: indicate the differential microorganisms that coincide with the LEfSe analysis.

**Table S3 Different microorganisms in the gastric mucosa of Cirrhosis A compared to Cirrhosis N and Control respectively**

| **Microorganisms** | ***P*-adjust** | |
| --- | --- | --- |
|  | **Cirrhosis A vs Cirrhosis N** | **Cirrhosis A vs Control** |
| **Genus** | | |
| *g__Actinobacillus* | 0.04665 | NS |
| *g__Lachnoanaerobaculum** | 0.04143 | 0.00036 |
| *g__Olsenella** | 0.04036 | 0.0000007 |
| *g__norank_f__norank_o__Subgroup_7* | 0.03690 | NS |
| *g__Allobaculum* | 0.02658 | NS |
| *g__Tepidimonas* | 0.03042 | NS |
| *g__Isoptericola* | 0.04079 | NS |
| *g__norank_f__norank_o__norank_c__MB-A2-108* | 0.04076 | NS |
| *g__Caulobacter** | 0.01580 | 0.01989 |
| *g__Marmoricola* | 0.02658 | NS |
| *g__norank_f__P5D1-392** | 0.02655 | 0.01321 |
| *g__Streptococcus** | NS | 0.00726 |
| *g__Prevotella** | NS | 0.00261 |
| *g__Neisseria** | NS | 0.00187 |
| *g__Veillonella** | NS | 0.00039 |
| *g__Fusobacterium** | NS | 0.00536 |
| *g__Haemophilus** | NS | 0.00665 |
| *g__Actinomyces** | NS | 0.00141 |
| *g__Alloprevotella** | NS | 0.03499 |
| *g__Porphyromonas** | NS | 0.00083 |
| *g__Granulicatella** | NS | 0.00520 |
| *g__Gemella** | NS | 0.00102 |
| *g__Rothia** | NS | 0.01412 |
| *g__Peptostreptococcus** | NS | 0.00142 |
| *g__Leptotrichia** | NS | 0.00142 |
| *g__Psychrobacter** | NS | 0.03325 |
| *g__Vagococcus** | NS | 0.03546 |
| *g__TM7x** | NS | 0.00023 |
| *g__Aggregatibacter** | NS | 0.00748 |
| *g__Solobacterium** | NS | 0.00032 |
| *g__Atopobium** | NS | 0.00008 |
| *g__Eubacterium_nodatum_group** | NS | 0.00149 |
| *g__Parvimonas** | NS | 0.00008 |
| *g__Campylobacter** | NS | 0.00147 |
| *g__norank_f__norank_o__Saccharimonadales** | NS | 0.01993 |
| *g__Capnocytophaga** | NS | 0.00520 |
| *g__Megasphaera** | NS | 0.00250 |
| *g__Dialister** | NS | 0.00001 |
| *g__Oribacterium** | NS | 0.00058 |
| *g__Stomatobaculum** | NS | 0.00257 |
| *g__Selenomonas** | NS | 0.00120 |
| *g__Treponema** | NS | 0.00188 |
| *g__Kurthia** | NS | 0.04413 |
| *g__Escherichia-Shigella** | NS | 0.02791 |
| *g__Filifactor** | NS | 0.00185 |
| *g__Catonella** | NS | 0.00001 |
| *g__Carnobacterium** | NS | 0.03736 |
| *g__Candidatus_Saccharimonas** | NS | 0.00786 |
| *g__Mogibacterium** | NS | 0.00020 |
| *g__norank_f__norank_o__Clostridia_UCG-014** | NS | 0.03972 |
| *g__Shewanella** | NS | 0.02334 |
| *g__Butyrivibrio** | NS | 0.02874 |
| *g__Brochothrix** | NS | 0.04299 |
| *g__Centipeda** | NS | 0.00053 |
| *g__Abiotrophia** | NS | 0.00021 |
| *g__Lautropia** | NS | 0.01412 |
| *g__F0332** | NS | 0.00051 |
| *g__Fretibacterium** | NS | 0.00276 |
| *g__norank_f__Saccharimonadaceae** | NS | 0.00208 |
| *g__Lysinibacillus** | NS | 0.00926 |
| *g__Bergeyella** | NS | 0.00882 |
| *g__Erysipelothrix** | NS | 0.00926 |
| *g__Tannerella** | NS | 0.04137 |
| *g__Morganella** | NS | 0.02918 |
| *g__Peptoanaerobacter** | NS | 0.00371 |
| *g__Eikenella** | NS | 0.00151 |
| *g__Johnsonella** | NS | 0.01327 |
| *g__norank_f__norank_o__norank_c__Gracilibacteria** | NS | 0.00466 |
| *g__norank_f__norank_o__Clostridia_vadinBB60_group** | NS | 0.00980 |
| *g__Eubacterium_yurii_group** | NS | 0.01741 |
| *g__Eubacterium_brachy_group** | NS | 0.01101 |
| *g__norank_f__norank_o__RF39** | NS | 0.03156 |
| *g__Fusicatenibacter** | NS | 0.04681 |
| *g__Kroppenstedtia** | NS | 0.03160 |
| *g__unclassified_f__Selenomonadaceae* | NS | 0.02915 |
| *g__Lentimicrobium* | NS | 0.00894 |
| *g__Eubacterium_saphenum_group** | NS | 0.04124 |
| *g__Rickettsia** | NS | 0.04671 |
| *g__Providencia** | NS | 0.03964 |
| *g__Pedobacter** | NS | 0.03160 |
| *g__Virgibacillus** | NS | 0.04718 |
| *g__Shuttleworthia* | NS | 0.00226 |
| *g__Anaeroglobus* | NS | 0.01087 |
| *g__norank_f__norank_o__RBG-13-54-9** | NS | 0.04671 |
| *g__norank_f__norank_o__Micavibrionales** | NS | 0.00371 |
| *g__Eggerthia* | NS | 0.02175 |
| *g__Pyramidobacter* | NS | 0.01323 |
| *g__Clostridium_sensu_stricto_13** | NS | 0.04671 |
| *g__Cryptobacterium** | NS | 0.01323 |
| *g__Slackia** | NS | 0.01719 |
| *g__Sediminibacterium* | NS | 0.04671 |
| *g__Scardovia* | NS | 0.00371 |
| *g__Pseudopropionibacterium* | NS | 0.00523 |
| *g__Defluviitaleaceae_UCG-011* | NS | 0.00760 |
| *g__Streptobacillus* | NS | 0.02737 |
| *g__Family_XIII_UCG-001* | NS | 0.02555 |
| *g__Howardella* | NS | 0.02443 |
| *g__Roseisolibacter** | NS | 0.04671 |
| *g__Parascardovia* | NS | 0.02735 |
| *g__Pseudoramibacter** | NS | 0.00101 |
| *g__Phocaeicola** | NS | 0.04671 |
| *g__norank_f__Weeksellaceae* | NS | 0.04671 |
| *g__norank_f__A0839** | NS | 0.04671 |
| *g__Pelospora** | NS | 0.04671 |

Red filling: indicates that the microorganism was significantly up-regulated in Cirrhosis A; Blue filling: indicates that the microorganism was significantly down-regulated in Cirrhosis A; Stars: indicate the differential microorganisms that coincide with the LEfSe analysis.

**Table S4 Top 100 DEGs between Cirrhosis A and Cirrhosis N in the gastric mucosa**

| **Gene name** | **Gene id** | **FC (Cirrhosis A/Control)** | **Log2FC**  **(Cirrhosis A/Control)** | ***P*-value** | ***P*-adjust** | **Regulate** |
| --- | --- | --- | --- | --- | --- | --- |
| REG1B | ENSG00000172023 | 40789993.24 | 25.28171193 | 2.97E-17 | 5.43E-13 | up |
| CARNS1* | ENSG00000172508 | 0.08396564 | -3.574057117 | 8.22E-13 | 7.51E-09 | down |
| MGAM2 | ENSG00000257743 | 20.26876337 | 4.341186165 | 1.87E-12 | 1.14E-08 | up |
| - | ENSG00000286037 | 20.12142218 | 4.330660373 | 1.39E-10 | 6.38E-07 | up |
| MAP3K20-AS1 | ENSG00000238133 | 16.7137168 | 4.062960691 | 4.31E-10 | 1.57E-06 | up |
| FLVCR1 | ENSG00000162769 | 2.277626763 | 1.18753135 | 6.50E-10 | 1.98E-06 | up |
| CFTR | ENSG00000001626 | 5.101393406 | 2.350891362 | 9.00E-10 | 2.35E-06 | up |
| SLC26A2 | ENSG00000155850 | 2.394751501 | 1.259875958 | 1.10E-09 | 2.51E-06 | up |
| XPR1 | ENSG00000143324 | 2.284845369 | 1.192096532 | 4.46E-09 | 9.07E-06 | up |
| IGFALS* | ENSG00000099769 | 0.055988094 | -4.158736126 | 7.91E-09 | 0.000014 | down |
| SLC5A1* | ENSG00000100170 | 14.64795351 | 3.872627213 | 0.000000013 | 0.000022 | up |
| CCR4 | ENSG00000183813 | 5.378314627 | 2.427154154 | 0.000000024 | 0.000036 | up |
| GCH1 | ENSG00000131979 | 2.132674357 | 1.092663694 | 0.000000026 | 0.000037 | up |
| TMEM236* | ENSG00000148483 | 15.58003441 | 3.961626515 | 0.000000028 | 0.000037 | up |
| ZNF579 | ENSG00000218891 | 0.483830497 | -1.047426385 | 0.000000031 | 0.000038 | down |
| PIK3AP1 | ENSG00000155629 | 2.238906709 | 1.162794415 | 0.000000185 | 0.000199 | up |
| DPP4* | ENSG00000197635 | 5.773648652 | 2.529483317 | 0.000000214 | 0.000218 | up |
| SLC19A3 | ENSG00000135917 | 5.719266361 | 2.515830097 | 0.000000233 | 0.000225 | up |
| HPS5 | ENSG00000110756 | 2.048537141 | 1.03459405 | 0.000000434 | 0.000378 | up |
| SLC49A3 | ENSG00000169026 | 0.460025143 | -1.120215381 | 0.000000472 | 0.000392 | down |
| PHYHIPL* | ENSG00000165443 | 13.09045911 | 3.710443791 | 0.000000585 | 0.000465 | up |
| C2orf74* | ENSG00000237651 | 0.322964548 | -1.630552286 | 0.000000850 | 0.000647 | down |
| LIPG* | ENSG00000101670 | 4.776884316 | 2.256069939 | 0.000001236 | 0.000869 | up |
| LPGAT1 | ENSG00000123684 | 2.338312576 | 1.225467796 | 0.000001388 | 0.000940 | up |
| CD320* | ENSG00000167775 | 0.356792834 | -1.486841455 | 0.000001899 | 0.001240 | down |
| LPAR3* | ENSG00000171517 | 4.470897118 | 2.160564348 | 0.000002121 | 0.001293 | up |
| CFAP73* | ENSG00000186710 | 0.241771748 | -2.048282426 | 0.000002095 | 0.001293 | down |
| MTNR1A* | ENSG00000168412 | 19.18802864 | 4.262134593 | 0.000002474 | 0.001414 | up |
| FAM20A | ENSG00000108950 | 0.491133534 | -1.025812765 | 0.000002700 | 0.001476 | down |
| CCR6 | ENSG00000112486 | 2.802665786 | 1.486799715 | 0.000002835 | 0.001476 | up |
| PLEKHG1* | ENSG00000120278 | 2.19287881 | 1.132826083 | 0.000002905 | 0.001476 | up |
| MGAM | ENSG00000257335 | 6.657293849 | 2.734935849 | 0.000002827 | 0.001476 | up |
| TMEM151A* | ENSG00000179292 | 0.189143673 | -2.402445577 | 0.000003642 | 0.001738 | down |
| LINC03040 | ENSG00000181577 | 8.587615888 | 3.102257663 | 0.000003706 | 0.001738 | up |
| SCG2 | ENSG00000171951 | 4.351724238 | 2.121587138 | 0.000004035 | 0.001829 | up |
| BAD | ENSG00000002330 | 0.474437124 | -1.075711192 | 0.000004503 | 0.001916 | down |
| SLC16A4* | ENSG00000168679 | 5.324046756 | 2.412523241 | 0.000004505 | 0.001916 | up |
| PIGR* | ENSG00000162896 | 5.179767924 | 2.37288746 | 0.000004927 | 0.002002 | up |
| CP | ENSG00000047457 | 9.20543668 | 3.202486161 | 0.000005290 | 0.002058 | up |
| - | ENSG00000279117 | 0.463391709 | -1.109695863 | 0.000005211 | 0.002058 | down |
| TYRP1 | ENSG00000107165 | 6.00013604 | 2.584995211 | 0.000005549 | 0.002114 | up |
| CCL20 | ENSG00000115009 | 17.11852759 | 4.097486712 | 0.000005783 | 0.002158 | up |
| NHERF2 | ENSG00000065054 | 0.49488216 | -1.014843061 | 0.000006059 | 0.002216 | down |
| PSMG4 | ENSG00000180822 | 0.416496342 | -1.263624269 | 0.000006198 | 0.002222 | down |
| SLC6A4 | ENSG00000108576 | 8.266084102 | 3.047204042 | 0.000006553 | 0.002305 | up |
| BAIAP2 | ENSG00000175866 | 0.461821892 | -1.114591531 | 0.000006958 | 0.002401 | down |
| CPS1* | ENSG00000021826 | 44.94984502 | 5.490244237 | 0.000007176 | 0.002430 | up |
| LY75 | ENSG00000054219 | 2.112746768 | 1.079119857 | 0.000007552 | 0.002511 | up |
| MYB* | ENSG00000118513 | 3.007853272 | 1.588734192 | 0.000008625 | 0.002719 | up |
| RGL3* | ENSG00000205517 | 0.412770141 | -1.276589483 | 0.000008522 | 0.002719 | down |
| PRDM16-DT* | ENSG00000177133 | 0.190707022 | -2.390570127 | 0.000009720 | 0.003013 | down |
| SLC26A9* | ENSG00000174502 | 0.287641619 | -1.797655658 | 0.000010055 | 0.003065 | down |
| MMP7 | ENSG00000137673 | 5.70670681 | 2.512658445 | 0.000010740 | 0.003177 | up |
| KLHL23 | ENSG00000213160 | 2.249087937 | 1.16934007 | 0.000014049 | 0.003953 | up |
| MROH7* | ENSG00000184313 | 0.267554361 | -1.902096051 | 0.000014395 | 0.003989 | down |
| ZNF524 | ENSG00000171443 | 0.470151071 | -1.088803691 | 0.000015447 | 0.004154 | down |
| GFOD1 | ENSG00000145990 | 2.06619868 | 1.046978987 | 0.000016459 | 0.004362 | up |
| CDH3 | ENSG00000062038 | 4.05716879 | 2.020473324 | 0.000017640 | 0.004481 | up |
| CA9 | ENSG00000107159 | 0.245645372 | -2.025351033 | 0.000017508 | 0.004481 | down |
| GPD1* | ENSG00000167588 | 7.151789438 | 2.838304262 | 0.000017373 | 0.004481 | up |
| MSMO1* | ENSG00000052802 | 2.866612935 | 1.519347118 | 0.000018261 | 0.004534 | up |
| TP53I13* | ENSG00000167543 | 0.472390456 | -1.081948278 | 0.000018486 | 0.004534 | down |
| IGKV6-21 | ENSG00000211611 | 7.008439004 | 2.809093147 | 0.000018595 | 0.004534 | up |
| IGHG2 | ENSG00000211893 | 11.35182605 | 3.504852483 | 0.000018965 | 0.004564 | up |
| ABCB1 | ENSG00000085563 | 3.206048112 | 1.680796076 | 0.000019269 | 0.004577 | up |
| ENTPD7* | ENSG00000198018 | 2.463364927 | 1.300630367 | 0.000020151 | 0.004606 | up |
| GATA6-AS1* | ENSG00000266010 | 0.453172186 | -1.141868777 | 0.000021652 | 0.004829 | down |
| GPRC5C* | ENSG00000170412 | 0.347468191 | -1.525047183 | 0.000022784 | 0.005020 | down |
| PGAM4* | ENSG00000226784 | 21.60206762 | 4.4330975 | 0.000023116 | 0.005033 | up |
| DCXR | ENSG00000169738 | 0.405559074 | -1.30201602 | 0.000024288 | 0.005226 | down |
| ME3* | ENSG00000151376 | 0.366747458 | -1.44714113 | 0.000026387 | 0.005611 | down |
| PWWP2B* | ENSG00000171813 | 0.432814489 | -1.208179299 | 0.000027651 | 0.005746 | down |
| SLC30A10* | ENSG00000196660 | 11.27267522 | 3.49475803 | 0.000027481 | 0.005746 | up |
| CXCL2 | ENSG00000081041 | 6.436425927 | 2.686259799 | 0.000031118 | 0.006015 | up |
| CST3 | ENSG00000101439 | 0.49677082 | -1.00934766 | 0.000030314 | 0.006015 | down |
| EMB | ENSG00000170571 | 2.269127628 | 1.182137756 | 0.000030117 | 0.006015 | up |
| FRMD5* | ENSG00000171877 | 0.460279434 | -1.119418113 | 0.000031244 | 0.006015 | down |
| SMIM10L2A* | ENSG00000178947 | 0.420024021 | -1.251456257 | 0.000030761 | 0.006015 | down |
| EPOR* | ENSG00000187266 | 0.430409861 | -1.216216963 | 0.000033309 | 0.006345 | down |
| CYP4F22* | ENSG00000171954 | 0.182721276 | -2.452283463 | 0.000033788 | 0.006370 | down |
| SLC2A5* | ENSG00000142583 | 20.6414055 | 4.367469304 | 0.000038356 | 0.007085 | up |
| PDZK1 | ENSG00000174827 | 8.583311697 | 3.10153439 | 0.000041380 | 0.007419 | up |
| DAB1 | ENSG00000173406 | 5.284117959 | 2.401662673 | 0.000042134 | 0.007445 | up |
| LINC02604* | ENSG00000273142 | 0.410844198 | -1.283336702 | 0.000042337 | 0.007445 | down |
| LTF | ENSG00000012223 | 12.31873791 | 3.62278255 | 0.000049747 | 0.008269 | up |
| TNFSF18* | ENSG00000120337 | 8.954791456 | 3.162659834 | 0.000050188 | 0.008269 | up |
| PNPLA7 | ENSG00000130653 | 0.356089397 | -1.489688615 | 0.000049653 | 0.008269 | down |
| DLGAP1 | ENSG00000170579 | 8.64701936 | 3.112202919 | 0.000049313 | 0.008269 | up |
| CFAP65* | ENSG00000181378 | 0.215922124 | -2.211417021 | 0.000050189 | 0.008269 | down |
| CNDP1* | ENSG00000150656 | 16.16482674 | 4.014786139 | 0.000051634 | 0.008373 | up |
| TFF2 | ENSG00000160181 | 0.375151941 | -1.414453071 | 0.000051735 | 0.008373 | down |
| HTR4 | ENSG00000164270 | 43.54294136 | 5.444366961 | 0.000052800 | 0.008470 | up |
| RNF223 | ENSG00000237330 | 0.360216692 | -1.473063061 | 0.000056237 | 0.008866 | down |
| ITGB8 | ENSG00000105855 | 2.349611234 | 1.232422069 | 0.000056764 | 0.008873 | up |
| QPCT | ENSG00000115828 | 3.838935752 | 1.940706416 | 0.000058862 | 0.009123 | up |
| FAM153CP* | ENSG00000204677 | 0.455921381 | -1.133143026 | 0.000061232 | 0.009410 | down |
| RIMS1* | ENSG00000079841 | 2.102150492 | 1.071865955 | 0.000062683 | 0.009441 | up |
| TRABD2A | ENSG00000186854 | 4.097440431 | 2.034722975 | 0.000062304 | 0.009441 | up |
| KLK11* | ENSG00000167757 | 0.305697254 | -1.709824502 | 0.000067040 | 0.009654 | down |
| COLGALT2 | ENSG00000198756 | 3.674546384 | 1.877566163 | 0.000066721 | 0.009654 | up |

**Stars**: indicate the same genes in both of the two graphs（indicate Cirrhosis A VS Control and Cirrhosis A VS Cirrhosis N） above.

**Table S5 KEGG enrichment pathways of DEGs between Cirrhosis A and Cirrhosis N in the gastric mucosa**

| **Pathway description** | **Ratio_in_study** | **Ratio_in_pop** | **Rich factor** | ***P*-value** | ***P*-adjust** |
| --- | --- | --- | --- | --- | --- |
| Carbohydrate digestion and absorption* | 6/ 223 | 43/ 15002 | 0.13953 | 0.00004 | 0.00443 |
| Nitrogen metabolism* | 4/ 223 | 17/ 15002 | 0.23529 | 0.00010 | 0.00554 |
| PPAR signaling pathway* | 7/ 223 | 71/ 15002 | 0.09859 | 0.00009 | 0.00658 |
| Bile secretion* | 8/ 223 | 85/ 15002 | 0.09412 | 0.00004 | 0.00863 |
| ABC transporters | 5/ 223 | 42/ 15002 | 0.11905 | 0.00038 | 0.01720 |
| Maturity onset diabetes of the young | 4/ 223 | 26/ 15002 | 0.15385 | 0.00055 | 0.02088 |
| Cholesterol metabolism* | 5/ 223 | 49/ 15002 | 0.10204 | 0.00078 | 0.02531 |
| Galactose metabolism | 4/ 223 | 31/ 15002 | 0.12903 | 0.00109 | 0.03110 |
| Mineral absorption* | 5/ 223 | 58/ 15002 | 0.08621 | 0.00168 | 0.03821 |
| Tyrosine metabolism | 4/ 223 | 36/ 15002 | 0.11111 | 0.00193 | 0.03996 |
| Rheumatoid arthritis | 6/ 223 | 85/ 15002 | 0.07059 | 0.00166 | 0.04207 |
| Steroid biosynthesis | 3/ 223 | 18/ 15002 | 0.16667 | 0.00224 | 0.04260 |

**Stars**: indicate the same transcripts in both of the two graphs（indicate Cirrhosis A VS Control and Cirrhosis A VS Cirrhosis N） above.

**Table S6 DO enrichment of DEGs between Cirrhosis A and Cirrhosis N in the gastric mucosa**

| **DO name** | **Ratio_in_study** | **Ratio_in_pop** | **Rich factor** | ***P*-value** | ***P*-adjust** |
| --- | --- | --- | --- | --- | --- |
| inherited metabolic disorder* | 58/ 158 | 1713/ 9251 | 0.03386 | 0.00000004 | 0.00004 |
| disease of metabolism* | 62/ 158 | 1987/ 9251 | 0.03120 | 0.00000025 | 0.00013 |
| vascular disease* | 53/ 158 | 1604/ 9251 | 0.03304 | 0.00000050 | 0.00017 |
| carbohydrate metabolic disorder* | 40/ 158 | 1058/ 9251 | 0.03781 | 0.00000076 | 0.00019 |
| glucose metabolism disease* | 37/ 158 | 967/ 9251 | 0.03826 | 0.00000167 | 0.00034 |
| Diarrhea | 7/ 158 | 43/ 9251 | 0.16279 | 0.00000714 | 0.00120 |
| gastrointestinal system disease* | 63/ 158 | 2321/ 9251 | 0.02714 | 0.00002661 | 0.00245 |
| Overnutrition* | 24/ 158 | 561/ 9251 | 0.04278 | 0.00002607 | 0.00264 |
| Obesity* | 24/ 158 | 558/ 9251 | 0.04301 | 0.00002389 | 0.00269 |
| diabetes mellitus* | 32/ 158 | 886/ 9251 | 0.03612 | 0.00003194 | 0.00269 |
| cardiovascular system disease | 58/ 158 | 2051/ 9251 | 0.02828 | 0.00001997 | 0.00289 |
| nutrition disease* | 25/ 158 | 595/ 9251 | 0.04202 | 0.00002350 | 0.00297 |
| artery disease* | 40/ 158 | 1256/ 9251 | 0.03185 | 0.00005049 | 0.00393 |
| Carcinoma* | 64/ 158 | 2430/ 9251 | 0.02634 | 0.00005905 | 0.00398 |
| acquired metabolic disease* | 26/ 158 | 666/ 9251 | 0.03904 | 0.00005573 | 0.00403 |
| Cholestasis* | 11/ 158 | 156/ 9251 | 0.07051 | 0.00007041 | 0.00445 |
| biliary tract disease* | 17/ 158 | 354/ 9251 | 0.04802 | 0.00010969 | 0.00483 |
| lipid storage disease* | 15/ 158 | 285/ 9251 | 0.05263 | 0.00010358 | 0.00499 |
| gastrointestinal system cancer* | 47/ 158 | 1628/ 9251 | 0.02887 | 0.00010946 | 0.00504 |
| Hypertension* | 29/ 158 | 824/ 9251 | 0.03519 | 0.00012731 | 0.00515 |
| bile duct disease* | 17/ 158 | 352/ 9251 | 0.04830 | 0.00010237 | 0.00518 |
| hepatobiliary disease* | 45/ 158 | 1526/ 9251 | 0.02949 | 0.00009824 | 0.00523 |
| intestinal disease* | 30/ 158 | 865/ 9251 | 0.03468 | 0.00012422 | 0.00524 |
| viral pneumonia | 3/ 158 | 6/ 9251 | 0.50000 | 0.00009415 | 0.00529 |
| disease by infectious agent | 35/ 158 | 1064/ 9251 | 0.03289 | 0.00008897 | 0.00530 |
| organ system cancer* | 83/ 158 | 3531/ 9251 | 0.02351 | 0.00015147 | 0.00568 |
| aspiration pneumonia | 3/ 158 | 7/ 9251 | 0.42857 | 0.00016270 | 0.00568 |
| Asthma* | 15/ 158 | 298/ 9251 | 0.05034 | 0.00016980 | 0.00573 |
| familial hyperlipidemia* | 9/ 158 | 115/ 9251 | 0.07826 | 0.00014780 | 0.00575 |
| Cholelithiasis* | 5/ 158 | 31/ 9251 | 0.16129 | 0.00016185 | 0.00585 |
| upper respiratory tract disease* | 15/ 158 | 300/ 9251 | 0.05000 | 0.00018271 | 0.00596 |
| pancreas disease* | 24/ 158 | 639/ 9251 | 0.03756 | 0.00020153 | 0.00637 |
| Cancer* | 109/ 158 | 5116/ 9251 | 0.02131 | 0.00027070 | 0.00830 |
| fatty liver disease* | 12/ 158 | 215/ 9251 | 0.05581 | 0.00030531 | 0.00858 |
| Sitosterolemia* | 2/ 158 | 2/ 9251 | 1.00000 | 0.00028989 | 0.00863 |
| bronchial disease | 15/ 158 | 314/ 9251 | 0.04777 | 0.00029947 | 0.00866 |
| respiratory system disease* | 40/ 158 | 1374/ 9251 | 0.02911 | 0.00036039 | 0.00986 |
| vitamin D-dependent rickets | 3/ 158 | 9/ 9251 | 0.33333 | 0.00038075 | 0.01014 |
| lysosomal storage disease* | 15/ 158 | 325/ 9251 | 0.04615 | 0.00043189 | 0.01121 |
| disease of cellular proliferation* | 109/ 158 | 5184/ 9251 | 0.02103 | 0.00052910 | 0.01339 |
| lower respiratory tract disease* | 37/ 158 | 1265/ 9251 | 0.02925 | 0.00058394 | 0.01441 |
| non-alcoholic fatty liver disease* | 8/ 158 | 114/ 9251 | 0.07018 | 0.00072157 | 0.01739 |
| exanthem | 2/ 158 | 3/ 9251 | 0.66667 | 0.00085988 | 0.01934 |
| cell type cancer* | 68/ 158 | 2867/ 9251 | 0.02372 | 0.00085155 | 0.01959 |
| hematopoietic system disease* | 43/ 158 | 1577/ 9251 | 0.02727 | 0.00083662 | 0.01969 |
| kidney disease* | 36/ 158 | 1256/ 9251 | 0.02866 | 0.00102764 | 0.02261 |
| type 2 diabetes mellitus* | 19/ 158 | 513/ 9251 | 0.03704 | 0.00115462 | 0.02486 |
| autoimmune disease* | 34/ 158 | 1174/ 9251 | 0.02896 | 0.00122312 | 0.02579 |
| colon cancer* | 15/ 158 | 365/ 9251 | 0.04110 | 0.00141767 | 0.02928 |
| Adenoma* | 15/ 158 | 365/ 9251 | 0.04110 | 0.00141767 | 0.02928 |
| lung disease* | 33/ 158 | 1146/ 9251 | 0.02880 | 0.00161214 | 0.03137 |
| eating disorder | 4/ 158 | 30/ 9251 | 0.13333 | 0.00158799 | 0.03151 |
| colonic disease | 16/ 158 | 412/ 9251 | 0.03883 | 0.00177129 | 0.03382 |
| Atherosclerosis* | 14/ 158 | 337/ 9251 | 0.04154 | 0.00184731 | 0.03462 |
| arteriosclerotic cardiovascular disease* | 14/ 158 | 338/ 9251 | 0.04142 | 0.00189902 | 0.03494 |
| endocrine system disease* | 40/ 158 | 1497/ 9251 | 0.02672 | 0.00198611 | 0.03589 |
| amino acid metabolic disorder | 8/ 158 | 135/ 9251 | 0.05926 | 0.00214941 | 0.03816 |
| Arteriosclerosis* | 15/ 158 | 382/ 9251 | 0.03927 | 0.00221403 | 0.03863 |
| viral infectious disease | 25/ 158 | 805/ 9251 | 0.03106 | 0.00237100 | 0.03934 |
| lipid metabolism disorder* | 9/ 158 | 168/ 9251 | 0.05357 | 0.00230717 | 0.03957 |
| rheumatoid arthritis | 19/ 158 | 546/ 9251 | 0.03480 | 0.00236808 | 0.03994 |
| liver disease* | 38/ 158 | 1421/ 9251 | 0.02674 | 0.00261465 | 0.04200 |
| nose disease | 9/ 158 | 171/ 9251 | 0.05263 | 0.00260119 | 0.04246 |
| pancreatitis | 9/ 158 | 172/ 9251 | 0.05233 | 0.00270555 | 0.04278 |
| gallbladder disease* | 7/ 158 | 111/ 9251 | 0.06306 | 0.00285681 | 0.04315 |
| acute necrotizing pancreatitis | 4/ 158 | 35/ 9251 | 0.11429 | 0.00283988 | 0.04354 |
| metachromatic leukodystrophy | 2/ 158 | 5/ 9251 | 0.40000 | 0.00280229 | 0.04363 |
| status epilepticus | 7/ 158 | 113/ 9251 | 0.06195 | 0.00315796 | 0.04566 |
| urinary system disease* | 37/ 158 | 1389/ 9251 | 0.02664 | 0.00322720 | 0.04600 |
| prostatitis | 3/ 158 | 18/ 9251 | 0.16667 | 0.00330361 | 0.04643 |
| bile duct adenocarcinoma* | 9/ 158 | 176/ 9251 | 0.05114 | 0.00315667 | 0.04698 |
| Cholangiocarcinoma* | 9/ 158 | 176/ 9251 | 0.05114 | 0.00315667 | 0.04698 |
| allergic disease* | 14/ 158 | 361/ 9251 | 0.03878 | 0.00346513 | 0.04739 |
| biliary tract cancer* | 11/ 158 | 248/ 9251 | 0.04435 | 0.00344971 | 0.04782 |

**Stars**: indicate the same transcripts in both of the two graphs（indicate Cirrhosis A VS Control and Cirrhosis A VS Cirrhosis N） above.

**Table S7 Top 100 DEGs between Cirrhosis A and Control A in the gastric mucosa**

| **Gene name** | **Gene id** | **FC**  **(Cirrhosis A/Control)** | **Log2FC**  **(Cirrhosis A/Control)** | ***P*-value** | ***P*-adjust** | **Regulate** |
| --- | --- | --- | --- | --- | --- | --- |
| ESM1 | ENSG00000164283 | 60.98177932 | 5.930306341 | 1.04E-25 | 2.06E-21 | up |
| SERPINE1 | ENSG00000106366 | 7.116080093 | 2.831082749 | 2.89E-18 | 2.87E-14 | up |
| CCN2 | ENSG00000118523 | 3.037272565 | 1.602776382 | 1.03E-16 | 6.81E-13 | up |
| APOB | ENSG00000084674 | 2262.379251 | 11.14362508 | 1.39E-13 | 6.89E-10 | up |
| CD320* | ENSG00000167775 | 0.274192041 | -1.8667414 | 1.78E-13 | 7.08E-10 | down |
| COL4A1 | ENSG00000187498 | 2.840156663 | 1.505970511 | 2.77E-13 | 9.15E-10 | up |
| VWF | ENSG00000110799 | 3.047275192 | 1.607519792 | 1.66E-10 | 3.34E-07 | up |
| RBP2 | ENSG00000114113 | 13803.94476 | 13.75279299 | 1.38E-10 | 3.34E-07 | up |
| MEP1B | ENSG00000141434 | 265.0241666 | 8.049980109 | 1.19E-10 | 3.34E-07 | up |
| CA4* | ENSG00000167434 | 0.080573897 | -3.633543651 | 1.69E-10 | 3.34E-07 | down |
| ANPEP | ENSG00000166825 | 114.8596521 | 6.843728287 | 2.39E-10 | 4.30E-07 | up |
| - | ENSG00000255639 | 4.780294584 | 2.257099527 | 3.43E-10 | 5.67E-07 | up |
| MTTP | ENSG00000138823 | 279.6363376 | 8.127408035 | 4.21E-10 | 6.42E-07 | up |
| IGFALS* | ENSG00000099769 | 0.076452961 | -3.709283804 | 2.92E-09 | 4.14E-06 | down |
| - | ENSG00000286037 | 25.83511451 | 4.691261373 | 5.06E-09 | 6.69E-06 | up |
| SLC14A1 | ENSG00000141469 | 0.228159746 | -2.131883812 | 6.37E-09 | 7.43E-06 | down |
| CYP3A4 | ENSG00000160868 | 182.5520983 | 7.512164441 | 6.23E-09 | 7.43E-06 | up |
| ATP1B3 | ENSG00000069849 | 3.363864166 | 1.75011945 | 9.55E-09 | 0.000011 | up |
| MALL | ENSG00000144063 | 8.338988562 | 3.059872409 | 1.31E-08 | 0.000014 | up |
| LDHA | ENSG00000134333 | 2.644733085 | 1.403122128 | 1.48E-08 | 0.000014 | up |
| GBA3 | ENSG00000249948 | 165.8425047 | 7.37367 | 1.48E-08 | 0.000014 | up |
| C17orf78 | ENSG00000278505 | 886.3161019 | 9.791677513 | 1.76E-08 | 0.000016 | up |
| TRIM8-DT | ENSG00000272933 | 9.835267276 | 3.297964259 | 2.42E-08 | 0.000021 | up |
| SPINK4* | ENSG00000122711 | 185.8880501 | 7.538290219 | 2.97E-08 | 0.000025 | up |
| MALRD1* | ENSG00000204740 | 128.1939355 | 7.002184204 | 9.09E-08 | 0.000072 | up |
| MUC20 | ENSG00000176945 | 2.973360756 | 1.572094515 | 0.00000015 | 0.000107 | up |
| ENTPD7* | ENSG00000198018 | 2.955077353 | 1.563195895 | 0.00000016 | 0.000115 | up |
| PIGR* | ENSG00000162896 | 5.621906445 | 2.491059446 | 0.00000021 | 0.000142 | up |
| MC1R | ENSG00000258839 | 0.366205932 | -1.449272936 | 0.00000022 | 0.000142 | down |
| TPK1* | ENSG00000196511 | 3.127673185 | 1.645089771 | 0.00000027 | 0.000168 | up |
| ANGPT2 | ENSG00000091879 | 4.574337665 | 2.193562865 | 0.00000037 | 0.000216 | up |
| SERPINB1 | ENSG00000021355 | 2.083754749 | 1.059185488 | 0.00000045 | 0.000228 | up |
| ANXA13 | ENSG00000104537 | 120.3065051 | 6.910570843 | 0.00000043 | 0.000228 | up |
| MTCH2 | ENSG00000109919 | 2.115633944 | 1.081090028 | 0.00000045 | 0.000228 | up |
| CHP2 | ENSG00000166869 | 114.5471012 | 6.839797139 | 0.00000044 | 0.000228 | up |
| TMEM236* | ENSG00000148483 | 9.716305606 | 3.280407868 | 0.00000051 | 0.000254 | up |
| APLN | ENSG00000171388 | 10.15709554 | 3.344416012 | 0.00000060 | 0.000291 | up |
| - | ENSG00000188897 | 0.216319776 | -2.208762534 | 0.00000062 | 0.000293 | down |
| CARNS1* | ENSG00000172508 | 0.146989733 | -2.766212705 | 0.00000075 | 0.000338 | down |
| YJEFN3 | ENSG00000250067 | 0.325731875 | -1.618243189 | 0.00000080 | 0.000345 | down |
| GABRD | ENSG00000187730 | 5.649107199 | 2.498022878 | 0.00000082 | 0.000346 | up |
| GPA33 | ENSG00000143167 | 15.49623576 | 3.953845904 | 0.00000084 | 0.000348 | up |
| ZNF135 | ENSG00000176293 | 0.416160433 | -1.264788289 | 0.00000087 | 0.000350 | down |
| ALDOB | ENSG00000136872 | 44.23242856 | 5.46703255 | 0.00000096 | 0.000376 | up |
| LRRC19* | ENSG00000184434 | 31.16950665 | 4.962063415 | 0.00000097 | 0.000376 | up |
| CYP2C8 | ENSG00000138115 | 0.085292661 | -3.551434586 | 0.00000099 | 0.000376 | down |
| CASTOR1* | ENSG00000239282 | 0.342335599 | -1.546516772 | 0.00000102 | 0.000383 | down |
| TSPOAP1 | ENSG00000005379 | 0.388576509 | -1.363729407 | 0.00000114 | 0.000419 | down |
| APOA1* | ENSG00000118137 | 72.59243792 | 6.181747363 | 0.00000124 | 0.000440 | up |
| MYO7B | ENSG00000169994 | 63.99003816 | 5.999775422 | 0.00000124 | 0.000440 | up |
| AGMAT | ENSG00000116771 | 3.009185686 | 1.589373133 | 0.00000130 | 0.000451 | up |
| CCK | ENSG00000187094 | 9.499353224 | 3.247829289 | 0.00000162 | 0.000534 | up |
| SLC34A2 | ENSG00000157765 | 20.58261081 | 4.363354088 | 0.00000169 | 0.000550 | up |
| SLC22A18AS | ENSG00000254827 | 3.629565283 | 1.859796765 | 0.00000200 | 0.000639 | up |
| FGFBP1 | ENSG00000137440 | 6.734415731 | 2.751552785 | 0.00000219 | 0.000689 | up |
| NPW* | ENSG00000183971 | 0.167170952 | -2.580603915 | 0.00000225 | 0.000697 | down |
| ABCG5* | ENSG00000138075 | 37.09596903 | 5.213190522 | 0.00000244 | 0.000744 | up |
| SI | ENSG00000090402 | 394.8174725 | 8.625042027 | 0.00000258 | 0.000753 | up |
| LRRC3B | ENSG00000179796 | 0.139745987 | -2.839121236 | 0.00000255 | 0.000753 | down |
| - | ENSG00000286600 | 301.9030416 | 8.237941482 | 0.00000258 | 0.000753 | up |
| CLDN7 | ENSG00000181885 | 13.52296752 | 3.757339871 | 0.00000270 | 0.000774 | up |
| ALPI | ENSG00000163295 | 416.3097028 | 8.701513373 | 0.00000298 | 0.000821 | up |
| APOA4 | ENSG00000110244 | 1389.595604 | 10.44044938 | 0.00000315 | 0.000855 | up |
| FABP1 | ENSG00000163586 | 142.5160375 | 7.154980466 | 0.00000340 | 0.000911 | up |
| GCLM | ENSG00000023909 | 2.320789041 | 1.214615388 | 0.00000364 | 0.000961 | up |
| SCD* | ENSG00000099194 | 3.838224884 | 1.940439242 | 0.00000417 | 0.001061 | up |
| CHAC1 | ENSG00000128965 | 0.177069645 | -2.497611184 | 0.00000417 | 0.001061 | down |
| - | ENSG00000290018 | 9.818080848 | 3.295441047 | 0.00000412 | 0.001061 | up |
| SLC5A1* | ENSG00000100170 | 12.64065911 | 3.659999785 | 0.00000445 | 0.001104 | up |
| CPS1* | ENSG00000021826 | 42.22748471 | 5.40011041 | 0.00000456 | 0.001116 | up |
| SCN4A | ENSG00000007314 | 0.183323808 | -2.447533935 | 0.00000464 | 0.001122 | down |
| CDH17 | ENSG00000079112 | 31.10737559 | 4.95918478 | 0.00000494 | 0.001180 | up |
| MROH7* | ENSG00000184313 | 0.238928152 | -2.065351244 | 0.00000503 | 0.001187 | down |
| ITGA2* | ENSG00000164171 | 2.44819878 | 1.291720702 | 0.00000510 | 0.001190 | up |
| CDC42EP5 | ENSG00000167617 | 2.442719806 | 1.288488388 | 0.00000563 | 0.001297 | up |
| CFAP73* | ENSG00000186710 | 0.310432887 | -1.687646689 | 0.00000576 | 0.001314 | down |
| CDX2 | ENSG00000165556 | 11.66755142 | 3.54442992 | 0.00000595 | 0.001337 | up |
| MOCOS | ENSG00000075643 | 2.727157386 | 1.447397961 | 0.00000645 | 0.001421 | up |
| HMGCR | ENSG00000113161 | 2.029237838 | 1.020937967 | 0.00000664 | 0.001446 | up |
| RRP7BP | ENSG00000291035 | 0.476014593 | -1.070922294 | 0.00000678 | 0.001460 | down |
| GPD1* | ENSG00000167588 | 10.87340117 | 3.442731377 | 0.00000704 | 0.001485 | up |
| PTPN5 | ENSG00000110786 | 0.105326662 | -3.247057414 | 0.00000754 | 0.001548 | down |
| SLC6A19* | ENSG00000174358 | 28.99560462 | 4.857762317 | 0.00000783 | 0.001548 | up |
| C3orf85 | ENSG00000241224 | 526.64958 | 9.040699536 | 0.00000768 | 0.001548 | up |
| PKD1P5 | ENSG00000254681 | 0.302935855 | -1.722915752 | 0.00000747 | 0.001548 | down |
| RIMS1* | ENSG00000079841 | 3.430682101 | 1.778495447 | 0.00000811 | 0.001561 | up |
| GCNT3 | ENSG00000140297 | 3.474931425 | 1.796984508 | 0.00000819 | 0.001561 | up |
| FABP2 | ENSG00000145384 | 272.4629882 | 8.089916455 | 0.00000808 | 0.001561 | up |
| RGL3* | ENSG00000205517 | 0.393918061 | -1.344032529 | 0.00000833 | 0.001561 | down |
| CDK3 | ENSG00000250506 | 0.424853114 | -1.234963955 | 0.00000835 | 0.001561 | down |
| - | ENSG00000266086 | 0.455700985 | -1.133840606 | 0.00000858 | 0.001590 | down |
| ACE2 | ENSG00000130234 | 12.21343687 | 3.610397328 | 0.00000915 | 0.001680 | up |
| CALML4 | ENSG00000129007 | 2.753457754 | 1.461244473 | 0.00000961 | 0.001748 | up |
| - | ENSG00000282416 | 0.334564723 | -1.579642761 | 0.00001046 | 0.001885 | down |
| SLC51A | ENSG00000163959 | 15.11283725 | 3.917702629 | 0.00001109 | 0.001964 | up |
| CDC25A | ENSG00000164045 | 2.262221546 | 1.177740223 | 0.00001131 | 0.001966 | up |
| FILIP1 | ENSG00000118407 | 2.240216513 | 1.163638173 | 0.00001302 | 0.002224 | up |
| GADD45G* | ENSG00000130222 | 0.402361946 | -1.313434228 | 0.00001355 | 0.002295 | down |
| - | ENSG00000251615 | 0.456646073 | -1.130851668 | 0.00001371 | 0.002303 | down |
| SPSB3 | ENSG00000162032 | 0.485490999 | -1.042483547 | 0.00001401 | 0.002324 | down |

**Stars**: indicate the same transcripts in both of the two graphs（indicate Cirrhosis A VS Control A and Cirrhosis A VS Cirrhosis N） above.

**Table S8 KEGG enrichment pathways of DEGs between Cirrhosis A and Control A in the gastric mucosa**

| **Pathway description** | **Ratio_in_study** | **Ratio_in_pop** | **Rich factor** | ***P*-value** | ***P*-adjust** |
| --- | --- | --- | --- | --- | --- |
| Fat digestion and absorption | 13/ 436 | 42/ 15002 | 0.30952 | 1.05E-10 | 2.99E-08 |
| PPAR signaling pathway* | 13/ 436 | 71/ 15002 | 0.18310 | 0.00000011 | 0.000016 |
| Cholesterol metabolism* | 10/ 436 | 49/ 15002 | 0.20408 | 0.00000115 | 0.000083 |
| Bile secretion* | 13/ 436 | 85/ 15002 | 0.15294 | 0.00000097 | 0.000093 |
| Protein digestion and absorption | 13/ 436 | 99/ 15002 | 0.13131 | 0.00000563 | 0.000322 |
| Renin-angiotensin system | 6/ 436 | 21/ 15002 | 0.28571 | 0.00002180 | 0.001039 |
| Mineral absorption* | 9/ 436 | 58/ 15002 | 0.15517 | 0.00004099 | 0.001675 |
| Drug metabolism - other enzymes | 10/ 436 | 79/ 15002 | 0.12658 | 0.00009305 | 0.003327 |
| Vitamin digestion and absorption | 5/ 436 | 23/ 15002 | 0.21739 | 0.00044212 | 0.014050 |
| Arginine and proline metabolism | 7/ 436 | 49/ 15002 | 0.14286 | 0.00049902 | 0.014272 |
| Nitrogen metabolism* | 4/ 436 | 17/ 15002 | 0.23529 | 0.00123949 | 0.032227 |
| Cell cycle | 12/ 436 | 152/ 15002 | 0.07895 | 0.00162126 | 0.033120 |
| Carbohydrate digestion and absorption* | 6/ 436 | 43/ 15002 | 0.13953 | 0.00142655 | 0.033999 |
| Linoleic acid metabolism | 5/ 436 | 30/ 15002 | 0.16667 | 0.00158407 | 0.034850 |
| Small cell lung cancer | 8/ 436 | 79/ 15002 | 0.10127 | 0.00205074 | 0.039101 |

**Stars**: indicate the same transcripts in both of the two graphs（indicate Cirrhosis A VS Control A and Cirrhosis A VS Cirrhosis N） above.

**Table S9 DO enrichment of DEGs between Cirrhosis A and Control A in the gastric mucosa**

| **DO name** | **Ratio_in_study** | **Ratio_in_pop** | **Rich factor** | ***P-*value** | ***P*-adjust** |
| --- | --- | --- | --- | --- | --- |
| gastrointestinal system disease* | 138/ 331 | 2321/ 9251 | 0.05946 | 1.22E-11 | 1.75E-08 |
| biliary tract disease* | 37/ 331 | 354/ 9251 | 0.10452 | 3.43E-09 | 1.65E-06 |
| bile duct disease* | 37/ 331 | 352/ 9251 | 0.10511 | 2.93E-09 | 2.11E-06 |
| hepatobiliary disease* | 94/ 331 | 1526/ 9251 | 0.06160 | 2.24E-08 | 8.06E-06 |
| Cholelithiasis* | 10/ 331 | 31/ 9251 | 0.32258 | 6.82E-08 | 0.000010 |
| coronary artery disease | 44/ 331 | 522/ 9251 | 0.08429 | 7.69E-08 | 0.000010 |
| gastrointestinal system cancer* | 97/ 331 | 1628/ 9251 | 0.05958 | 6.62E-08 | 0.000011 |
| Carcinoma* | 130/ 331 | 2430/ 9251 | 0.05350 | 9.64E-08 | 0.000011 |
| kidney disease* | 80/ 331 | 1256/ 9251 | 0.06369 | 8.95E-08 | 0.000011 |
| endocrine gland cancer | 87/ 331 | 1399/ 9251 | 0.06219 | 6.03E-08 | 0.000011 |
| fatty liver disease* | 26/ 331 | 215/ 9251 | 0.12093 | 4.54E-08 | 0.000011 |
| glucose metabolism disease* | 67/ 331 | 967/ 9251 | 0.06929 | 5.78E-08 | 0.000012 |
| urinary system disease* | 87/ 331 | 1389/ 9251 | 0.06263 | 4.31E-08 | 0.000012 |
| hepatobiliary system cancer | 73/ 331 | 1116/ 9251 | 0.06541 | 1.36E-07 | 0.000014 |
| adenocarcinoma | 59/ 331 | 826/ 9251 | 0.07143 | 1.50E-07 | 0.000014 |
| diabetes mellitus* | 61/ 331 | 886/ 9251 | 0.06885 | 3.26E-07 | 0.000029 |
| carbohydrate metabolic disorder* | 69/ 331 | 1059/ 9251 | 0.06516 | 3.83E-07 | 0.000032 |
| inherited metabolic disorder* | 98/ 331 | 1714/ 9251 | 0.05718 | 4.24E-07 | 0.000034 |
| biliary tract cancer* | 26/ 331 | 248/ 9251 | 0.10484 | 7.86E-07 | 0.000060 |
| familial combined hyperlipidemia | 9/ 331 | 32/ 9251 | 0.28125 | 0.0000012 | 0.000080 |
| disease of metabolism* | 108/ 331 | 1988/ 9251 | 0.05433 | 0.0000011 | 0.000081 |
| Cholestasis* | 19/ 331 | 156/ 9251 | 0.12179 | 0.0000028 | 0.000182 |
| familial hyperlipidemia* | 16/ 331 | 115/ 9251 | 0.13913 | 0.0000030 | 0.000188 |
| lipid storage disease* | 27/ 331 | 285/ 9251 | 0.09474 | 0.0000035 | 0.000208 |
| cell type cancer* | 141/ 331 | 2867/ 9251 | 0.04918 | 0.0000038 | 0.000210 |
| bile duct cancer | 21/ 331 | 189/ 9251 | 0.11111 | 0.0000037 | 0.000214 |
| acquired metabolic disease* | 47/ 331 | 666/ 9251 | 0.07057 | 0.0000047 | 0.000249 |
| type 2 diabetes mellitus* | 39/ 331 | 513/ 9251 | 0.07602 | 0.0000059 | 0.000302 |
| artery disease* | 74/ 331 | 1256/ 9251 | 0.05892 | 0.0000063 | 0.000312 |
| liver disease* | 81/ 331 | 1421/ 9251 | 0.05700 | 0.0000072 | 0.000335 |
| disease of cellular proliferation* | 224/ 331 | 5184/ 9251 | 0.04321 | 0.0000070 | 0.000336 |
| bile duct carcinoma | 20/ 331 | 186/ 9251 | 0.10753 | 0.0000104 | 0.000469 |
| urinary system cancer | 46/ 331 | 667/ 9251 | 0.06897 | 0.0000110 | 0.000478 |
| non-alcoholic fatty liver disease* | 15/ 331 | 114/ 9251 | 0.13158 | 0.0000123 | 0.000521 |
| liver carcinoma | 62/ 331 | 1015/ 9251 | 0.06108 | 0.0000141 | 0.000549 |
| lysosomal storage disease* | 28/ 331 | 325/ 9251 | 0.08615 | 0.0000140 | 0.000560 |
| hepatocellular carcinoma | 62/ 331 | 1014/ 9251 | 0.06114 | 0.0000137 | 0.000563 |
| bile duct adenocarcinoma* | 19/ 331 | 176/ 9251 | 0.10795 | 0.0000164 | 0.000590 |
| Cholangiocarcinoma* | 19/ 331 | 176/ 9251 | 0.10795 | 0.0000164 | 0.000590 |
| Hypertension* | 53/ 331 | 824/ 9251 | 0.06432 | 0.0000162 | 0.000597 |
| nutrition disease* | 42/ 331 | 595/ 9251 | 0.07059 | 0.0000158 | 0.000599 |
| pancreas disease* | 44/ 331 | 639/ 9251 | 0.06886 | 0.0000181 | 0.000606 |
| Obesity* | 40/ 331 | 558/ 9251 | 0.07168 | 0.0000180 | 0.000615 |
| breast cancer | 49/ 331 | 744/ 9251 | 0.06586 | 0.0000190 | 0.000623 |
| thoracic cancer | 49/ 331 | 745/ 9251 | 0.06577 | 0.0000197 | 0.000631 |
| Overnutrition* | 40/ 331 | 561/ 9251 | 0.07130 | 0.0000203 | 0.000636 |
| liver cancer | 62/ 331 | 1028/ 9251 | 0.06031 | 0.0000210 | 0.000643 |
| diabetic retinopathy | 17/ 331 | 150/ 9251 | 0.11333 | 0.0000244 | 0.000731 |
| Cancer* | 219/ 331 | 5116/ 9251 | 0.04281 | 0.0000274 | 0.000804 |
| endocrine system disease* | 82/ 331 | 1497/ 9251 | 0.05478 | 0.0000282 | 0.000812 |
| renal carcinoma | 35/ 331 | 472/ 9251 | 0.07415 | 0.0000310 | 0.000858 |
| intestinal disease* | 54/ 331 | 865/ 9251 | 0.06243 | 0.0000309 | 0.000871 |
| glomerulosclerosis | 10/ 331 | 59/ 9251 | 0.16949 | 0.0000396 | 0.001017 |
| myocardial infarction | 32/ 331 | 421/ 9251 | 0.07601 | 0.0000428 | 0.001026 |
| renovascular hypertension | 7/ 331 | 28/ 9251 | 0.25000 | 0.0000436 | 0.001028 |
| thoracic disease | 49/ 331 | 765/ 9251 | 0.06405 | 0.0000394 | 0.001032 |
| Arteriosclerosis* | 30/ 331 | 382/ 9251 | 0.07853 | 0.0000409 | 0.001033 |
| breast disease | 49/ 331 | 764/ 9251 | 0.06414 | 0.0000381 | 0.001035 |
| gallbladder disease* | 14/ 331 | 111/ 9251 | 0.12613 | 0.0000389 | 0.001037 |
| renal hypertension | 9/ 331 | 48/ 9251 | 0.18750 | 0.0000421 | 0.001045 |
| secondary hypertension | 9/ 331 | 48/ 9251 | 0.18750 | 0.0000421 | 0.001045 |
| retinal vascular disease | 18/ 331 | 174/ 9251 | 0.10345 | 0.0000486 | 0.001127 |
| organ system cancer* | 161/ 331 | 3531/ 9251 | 0.04560 | 0.0000502 | 0.001146 |
| hypolipoproteinemia | 5/ 331 | 13/ 9251 | 0.38462 | 0.0000578 | 0.001299 |
| vascular disease* | 85/ 331 | 1604/ 9251 | 0.05299 | 0.0000657 | 0.001455 |
| Atherosclerosis* | 27/ 331 | 337/ 9251 | 0.08012 | 0.0000715 | 0.001558 |
| renal cell carcinoma | 32/ 331 | 433/ 9251 | 0.07390 | 0.0000735 | 0.001579 |
| arteriosclerotic cardiovascular disease* | 27/ 331 | 338/ 9251 | 0.07988 | 0.0000752 | 0.001590 |
| lipid metabolism disorder* | 17/ 331 | 168/ 9251 | 0.10119 | 0.0001036 | 0.002160 |
| chronic leukemia | 19/ 331 | 202/ 9251 | 0.09406 | 0.0001102 | 0.002266 |
| liver cirrhosis | 26/ 331 | 328/ 9251 | 0.07927 | 0.0001160 | 0.002352 |
| kidney cancer | 37/ 331 | 546/ 9251 | 0.06777 | 0.0001235 | 0.002467 |
| glomerulonephritis | 20/ 331 | 222/ 9251 | 0.09009 | 0.0001324 | 0.002609 |
| immune system disease | 86/ 331 | 1668/ 9251 | 0.05156 | 0.0001577 | 0.003067 |
| drug-induced hepatitis | 3/ 331 | 4/ 9251 | 0.75000 | 0.0001768 | 0.003392 |
| autoimmune disease of gastrointestinal tract | 23/ 331 | 282/ 9251 | 0.08156 | 0.0001898 | 0.003593 |
| breast carcinoma | 32/ 331 | 457/ 9251 | 0.07002 | 0.0002007 | 0.003751 |
| inflammatory bowel disease | 21/ 331 | 247/ 9251 | 0.08502 | 0.0002039 | 0.003762 |
| female reproductive system disease | 52/ 331 | 887/ 9251 | 0.05862 | 0.0002231 | 0.004063 |
| respiratory system disease* | 73/ 331 | 1374/ 9251 | 0.05313 | 0.0002348 | 0.004223 |
| benign neoplasm | 43/ 331 | 692/ 9251 | 0.06214 | 0.0002418 | 0.004295 |
| cell type benign neoplasm | 36/ 331 | 547/ 9251 | 0.06581 | 0.0002716 | 0.004766 |
| lung disease* | 63/ 331 | 1146/ 9251 | 0.05497 | 0.0002759 | 0.004783 |
| lower respiratory tract disease* | 68/ 331 | 1265/ 9251 | 0.05375 | 0.0002897 | 0.004847 |
| aortic valve stenosis | 7/ 331 | 37/ 9251 | 0.18919 | 0.0002872 | 0.004920 |
| abdominal obesity-metabolic syndrome | 7/ 331 | 37/ 9251 | 0.18919 | 0.0002872 | 0.004920 |
| autoimmune disease* | 64/ 331 | 1174/ 9251 | 0.05451 | 0.0003089 | 0.005052 |
| abdominal obesity-metabolic syndrome 1 | 6/ 331 | 27/ 9251 | 0.22222 | 0.0003138 | 0.005074 |
| focal segmental glomerulosclerosis | 8/ 331 | 49/ 9251 | 0.16327 | 0.0003087 | 0.005106 |
| pulmonary hypertension | 18/ 331 | 203/ 9251 | 0.08867 | 0.0003470 | 0.005548 |
| parasitic infectious disease | 17/ 331 | 186/ 9251 | 0.09140 | 0.0003543 | 0.005602 |
| hepatic vascular disease | 9/ 331 | 64/ 9251 | 0.14063 | 0.0004181 | 0.006539 |
| hypobetalipoproteinemia | 3/ 331 | 5/ 9251 | 0.60000 | 0.0004302 | 0.006657 |
| pancreatic cancer | 31/ 331 | 458/ 9251 | 0.06769 | 0.0004558 | 0.006977 |
| nephritis | 22/ 331 | 281/ 9251 | 0.07829 | 0.0004614 | 0.006989 |
| colitis | 15/ 331 | 160/ 9251 | 0.09375 | 0.0005978 | 0.008961 |
| reproductive system disease | 74/ 331 | 1444/ 9251 | 0.05125 | 0.0006289 | 0.009330 |
| end stage renal disease | 14/ 331 | 147/ 9251 | 0.09524 | 0.0007729 | 0.011349 |
| hematopoietic system disease* | 79/ 331 | 1577/ 9251 | 0.05010 | 0.0007876 | 0.011448 |
| familial hypercholesterolemia | 6/ 331 | 32/ 9251 | 0.18750 | 0.0008262 | 0.011889 |
| aortic valve disease | 8/ 331 | 57/ 9251 | 0.14035 | 0.0008823 | 0.012570 |
| chronic kidney disease | 17/ 331 | 204/ 9251 | 0.08333 | 0.0010183 | 0.014227 |
| cerebral infarction | 16/ 331 | 186/ 9251 | 0.08602 | 0.0010162 | 0.014336 |
| upper respiratory tract disease* | 22/ 331 | 300/ 9251 | 0.07333 | 0.0011068 | 0.015314 |
| kidney failure | 24/ 331 | 340/ 9251 | 0.07059 | 0.0011326 | 0.015521 |
| morbid obesity | 7/ 331 | 47/ 9251 | 0.14894 | 0.0012912 | 0.017046 |
| endometriosis | 21/ 331 | 284/ 9251 | 0.07394 | 0.0012877 | 0.017157 |
| lung cancer | 43/ 331 | 751/ 9251 | 0.05726 | 0.0013196 | 0.017262 |
| Sitosterolemia* | 2/ 331 | 2/ 9251 | 1.00000 | 0.0012765 | 0.017329 |
| familial hypobetalipoproteinemia 2 | 2/ 331 | 2/ 9251 | 1.00000 | 0.0012765 | 0.017329 |
| urinary bladder cancer | 18/ 331 | 228/ 9251 | 0.07895 | 0.0013614 | 0.017337 |
| Barrett's esophagus | 6/ 331 | 35/ 9251 | 0.17143 | 0.0013521 | 0.017372 |
| bladder disease | 21/ 331 | 285/ 9251 | 0.07368 | 0.0013456 | 0.017444 |
| thyroid gland disease | 27/ 331 | 409/ 9251 | 0.06601 | 0.0015337 | 0.019360 |
| hyperglycemia | 15/ 331 | 176/ 9251 | 0.08523 | 0.0015892 | 0.019546 |
| hepatitis | 27/ 331 | 410/ 9251 | 0.06585 | 0.0015890 | 0.019712 |
| endometrial carcinoma | 12/ 331 | 124/ 9251 | 0.09677 | 0.0015761 | 0.019721 |
| varicose veins | 5/ 331 | 25/ 9251 | 0.20000 | 0.0016742 | 0.020417 |
| eye disease | 55/ 331 | 1040/ 9251 | 0.05288 | 0.0017718 | 0.021425 |
| uterine disease | 28/ 331 | 435/ 9251 | 0.06437 | 0.0018331 | 0.021800 |
| coronary stenosis | 6/ 331 | 37/ 9251 | 0.16216 | 0.0018237 | 0.021869 |
| parasitic protozoa infectious disease | 14/ 331 | 161/ 9251 | 0.08696 | 0.0018662 | 0.022012 |
| common cold | 5/ 331 | 26/ 9251 | 0.19231 | 0.0020129 | 0.023550 |
| thoracic benign neoplasm | 4/ 331 | 16/ 9251 | 0.25000 | 0.0020817 | 0.024157 |
| intestinal cancer | 34/ 331 | 569/ 9251 | 0.05975 | 0.0021542 | 0.024799 |
| demyelinating disease | 18/ 331 | 238/ 9251 | 0.07563 | 0.0021969 | 0.025090 |
| mixed cell type cancer | 3/ 331 | 8/ 9251 | 0.37500 | 0.0022231 | 0.025190 |
| primary immunodeficiency disease | 66/ 331 | 1317/ 9251 | 0.05011 | 0.0023351 | 0.026252 |
| retinal disease | 35/ 331 | 595/ 9251 | 0.05882 | 0.0024128 | 0.026708 |
| cerebral malaria | 5/ 331 | 27/ 9251 | 0.18519 | 0.0023991 | 0.026762 |
| allergic disease* | 24/ 331 | 361/ 9251 | 0.06648 | 0.0025277 | 0.027556 |
| esophageal disease | 21/ 331 | 300/ 9251 | 0.07000 | 0.0025215 | 0.027698 |
| portal hypertension | 7/ 331 | 53/ 9251 | 0.13208 | 0.0026352 | 0.028512 |
| malaria | 11/ 331 | 115/ 9251 | 0.09565 | 0.0026785 | 0.028764 |
| retinopathy of prematurity | 5/ 331 | 28/ 9251 | 0.17857 | 0.0028364 | 0.030234 |
| Adenoma* | 24/ 331 | 365/ 9251 | 0.06575 | 0.0029158 | 0.030851 |
| colon cancer* | 24/ 331 | 365/ 9251 | 0.06575 | 0.0029158 | 0.030851 |
| brain infarction | 16/ 331 | 207/ 9251 | 0.07729 | 0.0030638 | 0.031718 |
| multiple sclerosis | 17/ 331 | 226/ 9251 | 0.07522 | 0.0030549 | 0.031856 |
| premature menopause | 3/ 331 | 9/ 9251 | 0.33333 | 0.0032468 | 0.033372 |
| squamous cell carcinoma | 45/ 331 | 835/ 9251 | 0.05389 | 0.0033530 | 0.034219 |
| lung non-small cell carcinoma | 34/ 331 | 586/ 9251 | 0.05802 | 0.0034607 | 0.035070 |
| pre-eclampsia | 20/ 331 | 290/ 9251 | 0.06897 | 0.0037527 | 0.035762 |
| intracranial vasospasm | 2/ 331 | 3/ 9251 | 0.66667 | 0.0037386 | 0.037621 |
| xanthinuria | 2/ 331 | 3/ 9251 | 0.66667 | 0.0037386 | 0.037621 |
| cerebral creatine deficiency syndrome | 2/ 331 | 3/ 9251 | 0.66667 | 0.0037386 | 0.037621 |
| thyroid lymphoma | 2/ 331 | 3/ 9251 | 0.66667 | 0.0037386 | 0.037621 |
| von Willebrand's disease 1 | 2/ 331 | 3/ 9251 | 0.66667 | 0.0037386 | 0.037621 |
| Venezuelan equine encephalitis | 2/ 331 | 3/ 9251 | 0.66667 | 0.0037386 | 0.037621 |
| cystinuria | 2/ 331 | 3/ 9251 | 0.66667 | 0.0037386 | 0.037621 |
| paranasal sinus benign neoplasm | 2/ 331 | 3/ 9251 | 0.66667 | 0.0037386 | 0.037621 |
| pancreatic ductal carcinoma | 8/ 331 | 72/ 9251 | 0.11111 | 0.0040211 | 0.038068 |
| myeloid neoplasm | 22/ 331 | 333/ 9251 | 0.06607 | 0.0040685 | 0.038265 |
| lung carcinoma | 37/ 331 | 663/ 9251 | 0.05581 | 0.0044259 | 0.041356 |
| endometrial adenocarcinoma | 5/ 331 | 31/ 9251 | 0.16129 | 0.0044922 | 0.041705 |
| hematologic cancer | 63/ 331 | 1280/ 9251 | 0.04922 | 0.0045332 | 0.041815 |
| reproductive organ cancer | 50/ 331 | 967/ 9251 | 0.05171 | 0.0045821 | 0.041998 |
| hepatitis C | 17/ 331 | 236/ 9251 | 0.07203 | 0.0047452 | 0.042677 |
| bone marrow cancer | 23/ 331 | 358/ 9251 | 0.06425 | 0.0047046 | 0.042847 |
| respiratory system cancer | 43/ 331 | 804/ 9251 | 0.05348 | 0.0047427 | 0.042923 |
| aortic disease | 13/ 331 | 160/ 9251 | 0.08125 | 0.0048118 | 0.043007 |
| coronary restenosis | 4/ 331 | 20/ 9251 | 0.20000 | 0.0049497 | 0.043967 |
| intermediate coronary syndrome | 4/ 331 | 20/ 9251 | 0.20000 | 0.0049497 | 0.043967 |
| Asthma* | 20/ 331 | 298/ 9251 | 0.06711 | 0.0050825 | 0.044596 |
| bone marrow disease | 24/ 331 | 382/ 9251 | 0.06283 | 0.0051756 | 0.044865 |
| calcium metabolism disease | 5/ 331 | 32/ 9251 | 0.15625 | 0.0051711 | 0.045098 |
| sarcoidosis | 10/ 331 | 109/ 9251 | 0.09174 | 0.0055510 | 0.047831 |
| cerebrovascular disease | 30/ 331 | 515/ 9251 | 0.05825 | 0.0056042 | 0.048003 |

**Stars**: indicate the same transcripts in both of the two graphs（indicate Cirrhosis A VS Control A and Cirrhosis A VS Cirrhosis N） above.

**Table S10 Summary of sequencing quality control metrics for 16S rRNA amplicon sequencing**

| **SampleInfo** | **Seq_num** | **Base_num** | **Mean_length** | **Min_length** | **Max_length** |
| --- | --- | --- | --- | --- | --- |
| OC1 | 31629 | 13340590 | 421.78349 | 337 | 512 |
| OC2 | 61589 | 25945750 | 421.27247 | 238 | 540 |
| OC3 | 50118 | 21209401 | 423.18929 | 258 | 495 |
| OC4 | 59584 | 25214514 | 423.17592 | 211 | 473 |
| OC5 | 58765 | 24864451 | 423.11667 | 249 | 506 |
| OC6 | 48348 | 20376369 | 421.45216 | 354 | 507 |
| OC7 | 41479 | 17591616 | 424.10897 | 262 | 478 |
| OC8 | 49334 | 20809951 | 421.81763 | 222 | 482 |
| OC9 | 43774 | 18433432 | 421.10458 | 262 | 456 |
| OC10 | 49181 | 20597382 | 418.80771 | 245 | 515 |
| OC11 | 35472 | 14901165 | 420.08246 | 301 | 476 |
| OC12 | 40306 | 17071767 | 423.55399 | 270 | 432 |
| OC13 | 63693 | 26753019 | 420.03076 | 207 | 503 |
| OC14 | 49724 | 21030245 | 422.93953 | 221 | 476 |
| OC15 | 47705 | 20096430 | 421.26465 | 216 | 438 |
| OC16 | 38463 | 16235917 | 422.1178 | 235 | 462 |
| OC17 | 42995 | 18179013 | 422.81691 | 270 | 472 |
| OC18 | 35573 | 15176833 | 426.63911 | 262 | 480 |
| OC19 | 47641 | 20099417 | 421.89326 | 212 | 514 |
| OC20 | 42642 | 18100631 | 424.47894 | 270 | 482 |
| OC21 | 33408 | 14109070 | 422.32609 | 262 | 431 |
| OC22 | 36994 | 15691240 | 424.15635 | 262 | 435 |
| OC23 | 32354 | 13791696 | 426.27484 | 402 | 430 |
| OC24 | 33839 | 14353150 | 424.15999 | 262 | 431 |
| OC25 | 33276 | 14160582 | 425.54941 | 258 | 485 |
| OC26 | 38985 | 16528312 | 423.96594 | 270 | 477 |
| OC27 | 37947 | 15947554 | 420.25862 | 217 | 453 |
| OC28 | 34440 | 14612606 | 424.2917 | 318 | 494 |
| OC29 | 32338 | 13677652 | 422.95912 | 240 | 431 |
| OC30 | 43215 | 18438889 | 426.67798 | 235 | 430 |
| OC31 | 31541 | 13406919 | 425.06322 | 262 | 463 |
| OC32 | 39182 | 16610996 | 423.94457 | 270 | 463 |
| OC33 | 31786 | 13575201 | 427.08114 | 403 | 471 |
| OC34 | 116950 | 49618749 | 424.27319 | 261 | 506 |
| OC35 | 44725 | 18964605 | 424.02694 | 259 | 464 |
| OC36 | 31078 | 13201964 | 424.80095 | 262 | 431 |
| OC37 | 40864 | 17333218 | 424.16841 | 266 | 441 |
| OC38 | 34871 | 14631781 | 419.5974 | 271 | 461 |
| OC39 | 38533 | 16333895 | 423.89368 | 262 | 441 |
| OC40 | 35493 | 15028315 | 423.41631 | 232 | 458 |
| OC41 | 35158 | 14905986 | 423.97139 | 216 | 509 |
| OC42 | 32667 | 13839152 | 423.64319 | 265 | 479 |
| OC43 | 46200 | 19573795 | 423.67522 | 229 | 507 |
| OC44 | 44275 | 18658745 | 421.42846 | 251 | 507 |
| OC45 | 52557 | 22314680 | 424.58055 | 270 | 461 |
| OC46 | 36903 | 15596195 | 422.62675 | 387 | 432 |
| OC47 | 39632 | 16793575 | 423.73776 | 207 | 477 |
| OC48 | 33810 | 14275240 | 422.21946 | 215 | 461 |
| OC49 | 36368 | 15366972 | 422.54103 | 321 | 433 |
| OC50 | 46514 | 19768243 | 424.99555 | 270 | 507 |
| OC51 | 38743 | 16309107 | 420.95622 | 270 | 511 |
| OT1 | 51367 | 21702533 | 422.49952 | 262 | 500 |
| OT2 | 58412 | 24674762 | 422.42625 | 250 | 506 |
| OT3 | 51721 | 21822910 | 421.93519 | 227 | 462 |
| OT4 | 49086 | 20857998 | 424.92764 | 271 | 506 |
| OT5 | 58929 | 25016654 | 424.52195 | 320 | 508 |
| OT6 | 57976 | 24700010 | 426.03853 | 270 | 497 |
| OT7 | 62103 | 26246322 | 422.62567 | 253 | 506 |
| OT8 | 59075 | 24922215 | 421.87414 | 200 | 506 |
| OT9 | 58851 | 24900896 | 423.11764 | 270 | 507 |
| OT10 | 71509 | 30133374 | 421.39275 | 222 | 507 |
| OT11 | 64385 | 27234015 | 422.98695 | 205 | 520 |
| OT12 | 58253 | 24588225 | 422.09371 | 261 | 436 |
| OT13 | 62442 | 26417106 | 423.0663 | 237 | 516 |
| OT14 | 69847 | 29538514 | 422.90312 | 270 | 507 |
| OT15 | 63912 | 27021667 | 422.79489 | 245 | 438 |
| OT16 | 60294 | 25673851 | 425.81104 | 291 | 507 |
| OT17 | 68838 | 29036457 | 421.80855 | 337 | 435 |
| OT18 | 43231 | 18294681 | 423.18431 | 270 | 507 |
| OT19 | 40860 | 17306726 | 423.56158 | 277 | 506 |
| OT20 | 35211 | 15068270 | 427.94212 | 337 | 515 |
| OT21 | 42159 | 17855946 | 423.53818 | 261 | 507 |
| OT22 | 46049 | 19554238 | 424.6398 | 354 | 508 |
| OT23 | 45821 | 19328581 | 421.82801 | 337 | 508 |
| OT24 | 45178 | 19091273 | 422.57898 | 322 | 507 |
| OT25 | 41554 | 17486858 | 420.8225 | 337 | 437 |
| OT26 | 59233 | 25036318 | 422.67516 | 253 | 505 |
| OT27 | 59483 | 25124941 | 422.3886 | 259 | 506 |
| OT28 | 61009 | 25839173 | 423.53051 | 258 | 506 |
| OT29 | 58767 | 24545821 | 417.68035 | 218 | 507 |
| OT30 | 69874 | 29363735 | 420.23836 | 251 | 513 |
| OT31 | 60313 | 25514041 | 423.02723 | 343 | 466 |
| OT32 | 77358 | 32606927 | 421.50685 | 246 | 439 |
| OT33 | 49208 | 20892946 | 424.58434 | 237 | 507 |
| OT34 | 70469 | 29748272 | 422.14693 | 240 | 514 |
| OT35 | 68793 | 29172386 | 424.06038 | 245 | 497 |
| OT36 | 71726 | 30356852 | 423.23358 | 246 | 507 |
| OT37 | 62665 | 26631878 | 424.98808 | 213 | 520 |
| OT38 | 60307 | 25706791 | 426.26546 | 365 | 432 |
| OT39 | 68719 | 28880760 | 420.27329 | 245 | 506 |
| OT40 | 64863 | 27440419 | 423.05196 | 236 | 471 |
| OT41 | 72796 | 30833811 | 423.56463 | 245 | 507 |
| OT42 | 63973 | 26851350 | 419.72942 | 237 | 432 |
| OT43 | 34894 | 14633955 | 419.38313 | 290 | 510 |
| OT44 | 52096 | 22105172 | 424.31611 | 222 | 478 |
| OT45 | 39997 | 17000975 | 425.05625 | 261 | 442 |
| OT46 | 31139 | 13244805 | 425.34458 | 262 | 432 |
| OT47 | 50519 | 21252498 | 420.68327 | 223 | 485 |
| OT48 | 54324 | 22767609 | 419.10774 | 202 | 480 |
| OT49 | 34843 | 14499034 | 416.12473 | 266 | 461 |
| OT50 | 53300 | 22519042 | 422.4961 | 239 | 495 |
| OT51 | 49644 | 21034693 | 423.71068 | 218 | 433 |
| OT52 | 47392 | 20100617 | 424.13523 | 270 | 476 |
| OT53 | 50104 | 21176011 | 422.64113 | 262 | 507 |
| OT54 | 52859 | 22290670 | 421.70056 | 220 | 462 |
| OT55 | 51289 | 21583834 | 420.82774 | 250 | 455 |
| OT56 | 50424 | 21185912 | 420.15532 | 251 | 450 |
| OT57 | 53365 | 22642002 | 424.28562 | 245 | 470 |
| OT58 | 49921 | 21181918 | 424.30877 | 237 | 442 |
| OT59 | 53601 | 22545429 | 420.61583 | 270 | 476 |
| OT60 | 42387 | 18124043 | 427.58494 | 224 | 474 |
| OT61 | 48401 | 20530358 | 424.17219 | 270 | 472 |
| OT62 | 45505 | 19234963 | 422.69999 | 270 | 485 |
| OT63 | 47193 | 20042839 | 424.69941 | 261 | 462 |
| OT64 | 46150 | 19270377 | 417.55963 | 317 | 498 |
| OT65 | 47180 | 19931135 | 422.44881 | 220 | 487 |
| OT66 | 56987 | 24171533 | 424.15872 | 260 | 484 |
| OT67 | 51886 | 21958746 | 423.21139 | 202 | 503 |
| OT68 | 45772 | 19412084 | 424.10391 | 205 | 504 |
| OT69 | 47659 | 20245156 | 424.79188 | 254 | 507 |
| OT70 | 53494 | 22568186 | 421.88257 | 251 | 435 |
| OT71 | 53697 | 22521310 | 419.41468 | 211 | 510 |
| OT72 | 54660 | 23102382 | 422.65609 | 354 | 473 |
| OT73 | 54835 | 23090227 | 421.08557 | 264 | 506 |
| GC1 | 61656 | 25423191 | 412.33929 | 250 | 431 |
| GC2 | 54771 | 23353785 | 426.3896 | 200 | 532 |
| GC3 | 49775 | 20511104 | 412.07642 | 224 | 519 |
| GC4 | 46653 | 19678507 | 421.80582 | 254 | 462 |
| GC5 | 44939 | 18927784 | 421.18837 | 239 | 532 |
| GC6 | 40099 | 16235924 | 404.89598 | 249 | 517 |
| GC7 | 41049 | 16621661 | 404.92243 | 249 | 430 |
| GC8 | 36105 | 14480997 | 401.0801 | 214 | 433 |
| GC9 | 44343 | 18764050 | 423.15698 | 214 | 471 |
| GC10 | 39164 | 15849372 | 404.69237 | 249 | 470 |
| GC11 | 52775 | 22154386 | 419.78941 | 203 | 534 |
| GC12 | 42940 | 18196343 | 423.76206 | 249 | 514 |
| GC13 | 53741 | 22742307 | 423.18355 | 214 | 527 |
| GC14 | 59386 | 24068550 | 405.28997 | 250 | 473 |
| GC15 | 43548 | 18398239 | 422.48184 | 223 | 514 |
| GC16 | 39261 | 15858302 | 403.91997 | 246 | 430 |
| GC17 | 44035 | 18736247 | 425.48534 | 215 | 514 |
| GC18 | 32850 | 13275570 | 404.12694 | 201 | 521 |
| GC19 | 42445 | 18051956 | 425.3023 | 249 | 519 |
| GC20 | 37332 | 15835137 | 424.1706 | 249 | 509 |
| GC21 | 46030 | 18551390 | 403.02824 | 249 | 458 |
| GC22 | 43699 | 18640743 | 426.57139 | 249 | 519 |
| GC23 | 47574 | 20428554 | 429.40585 | 254 | 432 |
| GC24 | 46957 | 19910856 | 424.02317 | 214 | 517 |
| GC25 | 36225 | 14864530 | 410.33899 | 218 | 523 |
| GC26 | 73780 | 29757743 | 403.33075 | 240 | 471 |
| GC27 | 36441 | 14205674 | 389.82668 | 200 | 488 |
| GC28 | 43289 | 15620269 | 360.83691 | 200 | 516 |
| GC29 | 41217 | 16656846 | 404.12563 | 200 | 534 |
| GC30 | 51429 | 21943301 | 426.67174 | 219 | 480 |
| GT1 | 37321 | 15913946 | 426.40728 | 253 | 430 |
| GT2 | 40203 | 16214922 | 403.32617 | 208 | 518 |
| GT3 | 44969 | 19228978 | 427.6052 | 250 | 455 |
| GT4 | 40249 | 16755047 | 416.2848 | 200 | 529 |
| GT5 | 45195 | 18841717 | 416.89826 | 226 | 509 |
| GT6 | 57687 | 24408176 | 423.11398 | 250 | 508 |
| GT7 | 54469 | 22989517 | 422.06607 | 250 | 505 |
| GT8 | 63746 | 26693963 | 418.75511 | 207 | 532 |
| GT9 | 56967 | 23074727 | 405.05428 | 222 | 471 |
| GT10 | 54179 | 23016771 | 424.82827 | 249 | 490 |
| GT11 | 56591 | 23583542 | 416.73662 | 200 | 522 |
| GT12 | 57418 | 23336750 | 406.43614 | 249 | 445 |
| GT13 | 55165 | 23302915 | 422.4221 | 212 | 514 |
| GT14 | 45432 | 18725010 | 412.15465 | 209 | 525 |
| GT15 | 48999 | 20635408 | 421.13937 | 250 | 509 |
| GT16 | 55757 | 22740182 | 407.84443 | 226 | 456 |
| GT17 | 40673 | 17193790 | 422.73228 | 249 | 482 |
| GT18 | 56177 | 23789643 | 423.47657 | 249 | 492 |
| GT19 | 33015 | 13585642 | 411.49908 | 213 | 488 |
| GT20 | 32621 | 12386230 | 379.70111 | 200 | 514 |
| GT21 | 38297 | 15296490 | 399.41745 | 200 | 524 |
| GT22 | 280136 | 117267021 | 418.60747 | 201 | 536 |
| GT23 | 48587 | 20410999 | 420.09177 | 213 | 514 |
| GT24 | 32680 | 13802767 | 422.36129 | 248 | 519 |
| GT25 | 41117 | 16836084 | 409.46771 | 252 | 456 |
| GT26 | 39204 | 16549450 | 422.13677 | 201 | 484 |
| GT27 | 49631 | 20862527 | 420.35274 | 249 | 509 |
| GT28 | 44742 | 18931624 | 423.12869 | 249 | 517 |
| GT29 | 50191 | 21004551 | 418.49238 | 249 | 488 |
| GT30 | 57263 | 24189748 | 422.43243 | 249 | 485 |
| GT31 | 75462 | 30449871 | 403.51264 | 205 | 521 |
| GT32 | 52095 | 22092575 | 424.08245 | 249 | 514 |
| GT33 | 41217 | 16937667 | 410.93886 | 219 | 514 |
| GT34 | 35080 | 14660020 | 417.90251 | 245 | 481 |
| GT35 | 31228 | 12798222 | 409.83163 | 200 | 478 |
| GT36 | 51320 | 21516622 | 419.26387 | 223 | 526 |
| GT37 | 49599 | 20952636 | 422.44069 | 215 | 507 |
| GT38 | 49258 | 20764661 | 421.54901 | 207 | 532 |
| GT39 | 44752 | 18915341 | 422.67029 | 249 | 507 |
| **Total(193)** | **9616997** | **4040833065** | **420.02857958549** | **200** | **540** |
